# Increasing methylation of sperm rDNA and other repetitive elements in the aging male mammalian germline

**DOI:** 10.1111/acel.13181

**Published:** 2020-07-01

**Authors:** Ramya Potabattula, Federica Zacchini, Grazyna Ewa Ptak, Marcus Dittrich, Tobias Müller, Nady El Hajj, Thomas Hahn, Charis Drummer, Rüdiger Behr, Andrea Lucas‐Hahn, Heiner Niemann, Martin Schorsch, Thomas Haaf

**Affiliations:** ^1^ Institute of Human Genetics Julius Maximilians University Würzburg Germany; ^2^ Malopolska Centre of Biotechnology Jagiellonian University Krakow Poland; ^3^ Percuros B.V. Leiden The Netherlands; ^4^ Department of Bioinformatics Julius Maximilians University Würzburg Germany; ^5^ College of Health and Life Sciences Hamad Bin Khalifa University Doha Qatar; ^6^ Fertility Center Wiesbaden Germany; ^7^ Platform Degenerative Diseases Leibniz Institute for Primate Research Göttingen Germany; ^8^ German Center for Cardiovascular Research, Partner Site Göttingen Göttingen Germany; ^9^ Institute of Farm Animal Genetics Friedrich‐Loeffler‐Institute Mariensee/Neustadt Germany; ^10^ Clinic for Gastroenterology, Hepatology and Endocrinology Medical University Hannover Hannover Germany

**Keywords:** embryo developmental potential, epigenetic clock, germline aging, repetitive DNA elements, ribosomal DNA, sperm DNA methylation

## Abstract

In somatic cells/tissues, methylation of ribosomal DNA (rDNA) increases with age and age‐related pathologies, which has a direct impact on the regulation of nucleolar activity and cellular metabolism. Here, we used bisulfite pyrosequencing and show that methylation of the rDNA transcription unit including upstream control element (UCE), core promoter, 18S rDNA, and 28S rDNA in human sperm also significantly increases with donor's age. This positive correlation between sperm rDNA methylation and biological age is evolutionarily conserved among mammals with widely different life spans such as humans, marmoset, bovine, and mouse. Similar to the tandemly repeated rDNA, methylation of human α‐satellite and interspersed LINE1 repeats, marmoset α‐satellite, bovine alpha‐ and testis satellite I, mouse minor and major satellite, and LINE1‐T repeats increases in the aging male germline, probably related to their sperm histone packaging. Deep bisulfite sequencing of single rDNA molecules in human sperm revealed that methylation does not only depend on donor's age, but also depend on the region and sequence context (A vs. G alleles). Both average rDNA methylation of all analyzed DNA molecules and the number of fully (>50%) methylated alleles, which are thought to be epigenetically silenced, increase with donor's age. All analyzed CpGs in the sperm rDNA transcription unit show comparable age‐related methylation changes. Unlike other epigenetic aging markers, the rDNA clock appears to operate in similar ways in germline and soma in different mammalian species. We propose that sperm rDNA methylation, directly or indirectly, influences nucleolar formation and developmental potential in the early embryo.

## Graphical abstract

Sperm methylation of the rDNA transcription unit increases with donor's age in mammals with widely different life spans. Scatter plots show positive correlations between donor's age (*x*‐axis in percentage of life span) and mean methylation (*y*‐axis in %) of 18S rDNA in 80 mouse (purple dots), 36 bull (yellow), 16 marmoset (red), and 295 human (green) sperm samples. An orthologous region covering 8 CpGs was analyzed by bisulfite pyrosequencing. The black regression line applies to all 427 analyzed samples.

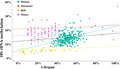

## 1 INTRODUCTION

Older men have a much lower chance of achieving a pregnancy by natural conception and assisted reproduction as well (Dain, Auslander, & Dirnfeld, 2011). Genome‐wide sequencing studies revealed an increasing rate of de novo genetic mutations in the offspring of older males (Kong et al., 2012), implying an elevated risk for rare monogenic (Crow, 2000) and neurodevelopmental disorders (Grether, Anderson, Croen, Smith, & Windham, 2009). The number of spermatogonial cell divisions prior to spermatogenesis increases from 35 times at puberty to >800 times at 50 years (Crow, 2000). During each replication cycle, not only the DNA sequence itself but also the epigenetic make‐up must be copied to the daughter cells. Since the error rate of this copying process is estimated to be 10–100 times higher for epigenetic than for genetic information (Bennett‐Baker, Wilkowski, & Burke, 2003), it can be assumed that the accumulation of epigenetic changes is critical for paternal aging. In the mouse model, age‐dependent sperm DNA methylation changes have been associated with changes in gene methylation and expression in the brain and abnormal behavior in the offspring of older males (Milekic et al., 2015). Age‐related sperm DNA methylation changes and their transmission to the next generation were also found in humans (Atsem et al., 2016; Jenkins, Aston, Pflueger, Cairns, & Carrell, 2014; Potabattula et al., 2018).

The association between aging and DNA methylation changes is very well documented across a broad spectrum of somatic tissues (Hannum et al., 2013; Horvath, 2013). In humans, epigenetic clocks which are built on methylation levels of highly selected CpGs can indicate (with error rates <5 years) the chronological age of the donor. DNA methylation age can also serve as a biomarker to predict life span and age‐related conditions (Field et al., 2018). The epigenetic signatures of the donor are erased and replaced by germline‐specific epigenomes in germ cells (Reik, Dean, & Walter, 2001). Because DNA methylation patterns largely differ between sperm and somatic cells (Molaro et al., 2011), epigenetic clocks that have been trained on somatic tissues cannot be used to predict the sperm donor's age. Overall, there is little overlap between the target CpGs of different epigenetic clocks which were all derived by linear regression algorithms on different data sets. The relationship (cause, consequence, or mere bystander) of clock CpGs to the aging process remains unclear (Field et al., 2018). The calculated DNA methylation age may be a surrogate marker that tracks the cumulative work done by an epigenetic maintenance system (Horvath, 2013) and an age‐dependent decay of the methylation landscape (Field et al., 2018).

Methylation‐dependent transcriptional regulation of ribosomal DNA (rDNA) is essential for ribosome biogenesis, protein synthesis, and each cellular process (Santoro & Grummt, 2001). Impaired ribosome biogenesis causes nucleolar stress that is involved in aging and pathogenesis of many age‐related diseases (Wang et al., 2015). In the human genome, several hundred rDNA transcription units are tandemly arrayed on the acrocentric short arms. Increased rDNA methylation with age has been observed in different rodent tissues (D'Aquila et al., 2017; Oakes, Smiraglia, Plass, Trasler, & Robaire, 2003) as well as during in vitro aging of human fibroblasts (Flunkert et al., 2018). Recently, an evolutionarily conserved epigenetic clock based on rDNA methylation has been developed on mouse blood methylation data sets (Wang & Lemos, 2019).

Apart from the tandemly arrayed rDNA transcription units, the human genome contains approximately 600,000 LINE‐1 and more than 1,000,000 ALU retrotransposons, comprising 17% and 11% of total genomic DNA, respectively (De Koning, Gu, Castoe, Batzer, & Pollock, 2011). Up to several megabases of α‐satellite DNA are present in the centromeric region of human chromosomes. Progressive loss of methylation in repetitive elements in somatic cells has been associated with aging and aging‐related diseases (Flunkert et al., 2018; Jones, Goodman, & Kobor, 2015).

## 2 RESULTS

### 2.1 Male aging effects on sperm repetitive DNA methylation

**FIGURE 1:**
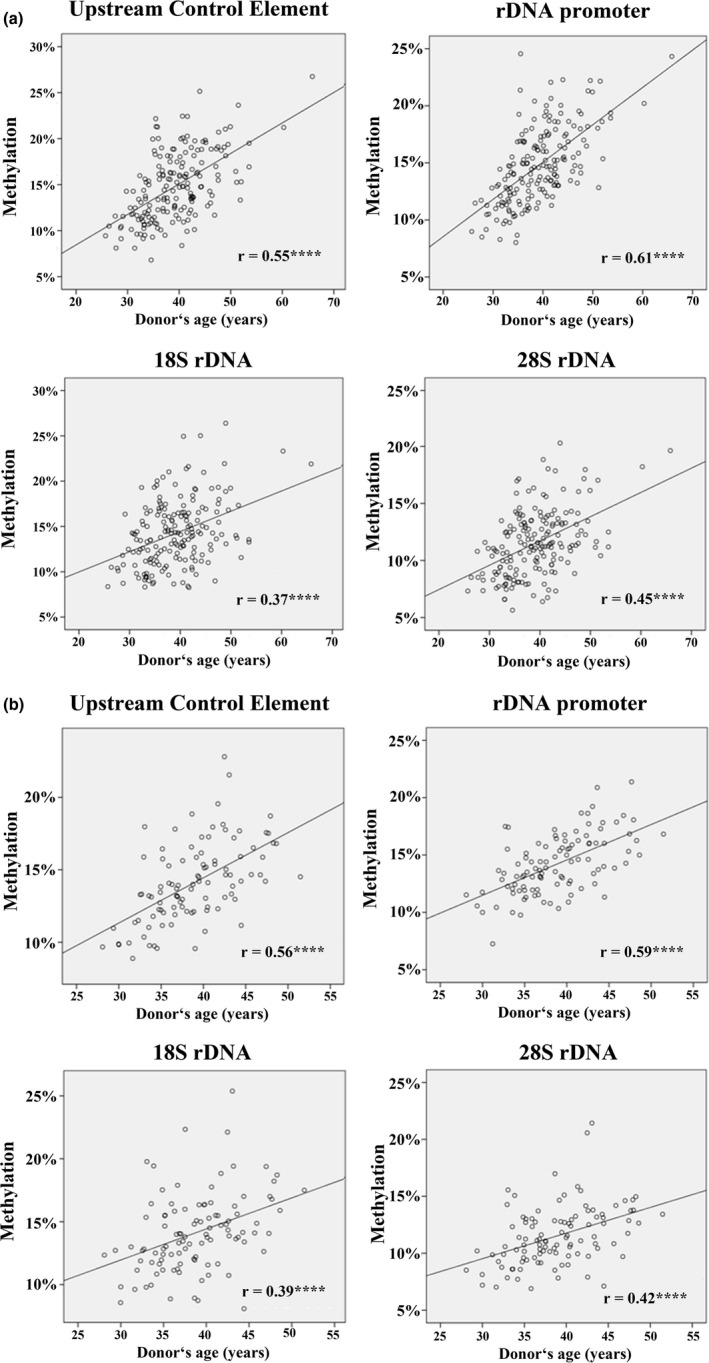
Methylation of the rDNA transcription unit in human sperm increases with donor's age. Scatter plots show significant (****p < 0.0001) positive correlations between donor's age (*x*‐axis in years) and mean methylation (*y*‐axis in %) of the UCE (26 CpGs), core promoter (9 CpGs), 18S rDNA (8 CpGs), and 28S rDNA (10 CpGs). One hundred and eighty‐six sperm samples of cohort 1 (a) and 109 samples of cohort 2 (b) were analyzed by bisulfite pyrosequencing. Pearson's partial correlations were used to adjust for confounding factors

Bisulfite pyrosequencing (BPS) was used to determine rDNA methylation levels of four target regions, including the upstream control element (UCE), core promoter, 18S rDNA, and 28S rDNA in human sperm samples of two independent cohorts, 1 (*N* = 186) and 2 (*N* = 109). Both cohorts displayed a wide range of donor's age (25–66 years), body mass index (BMI) (17–40 kg/m^2^), and sperm concentration (0.2–200 million/ml) (Table S1). The number of analyzed CpGs ranged from 8 in the 18S rDNA to 26 in the UCE amplicon (Table S2). Each of the 53 analyzed CpG sites in the rDNA transcription unit displayed a highly significant positive correlation with donor's age in both sperm cohorts (Table S3). Consequently, the mean sperm methylation of all four analyzed rDNA regions significantly (*p* < 0.0001) increased with donor's age (Figure 1). Pearson's partial correlations were applied to adjust mean methylation values for potential confounding factors such as sperm concentration and donor's BMI (Table 1).

**TABLE 1:** Pearson's partial correlations between donor's age and mean repeat methylation in human sperm cohorts 1 and 2

Region	Number of CpGs	Cohort 1 (*N* = 186)		Cohort 2 (*N* = 109)	
		Correlation coefficient	*p* Value	Correlation coefficient	*p* Value
rDNA upstream control element	26	+0.55	<0.0001	+0.56	<0.0001
rDNA promoter	9	+0.61	<0.0001	+0.59	<0.0001
18S rDNA	8	+0.37	<0.0001	+0.39	<0.0001
28S rDNA	10	+0.45	<0.0001	+0.42	<0.0001
α‐satellite DNA	4	+0.30	<0.0001	+0.28	0.007
LINE1	4	+0.28	<0.0001	+0.30	0.008
ALU	3	−0.05	0.47	−0.07	0.48

To test whether BPS also works reliably with very low sperm counts, eight genomic DNA samples were diluted down to aliquots equivalent to 10 sperm cells and analyzed by semi‐nested PCRs. Samples were pre‐selected to cover a maximum age range with 5‐year gaps. For most samples, the differences between three technical replicates were in the order of 2–3 percentage points. After adjusting for sperm concentration and donor's BMI, we observed significant Pearson's partial correlations between donor's age and rDNA (UCE and promoter) methylation as well (Figure S1).

In addition, BPS on sperm genomic DNA samples was used to determine methylation of other repetitive DNA families, including centromeric α‐satellite DNA and interspersed LINE1 and ALU repeats. For α‐satellite DNA and LINE1, there were highly significant positive correlations (both at the single CpG and the regional level) between sperm methylation and donor's age (Table 1 and Table S3).

### 2.2 Sequence‐ and age‐dependent rDNA methylation and epimutation rates

**FIGURE 2:**
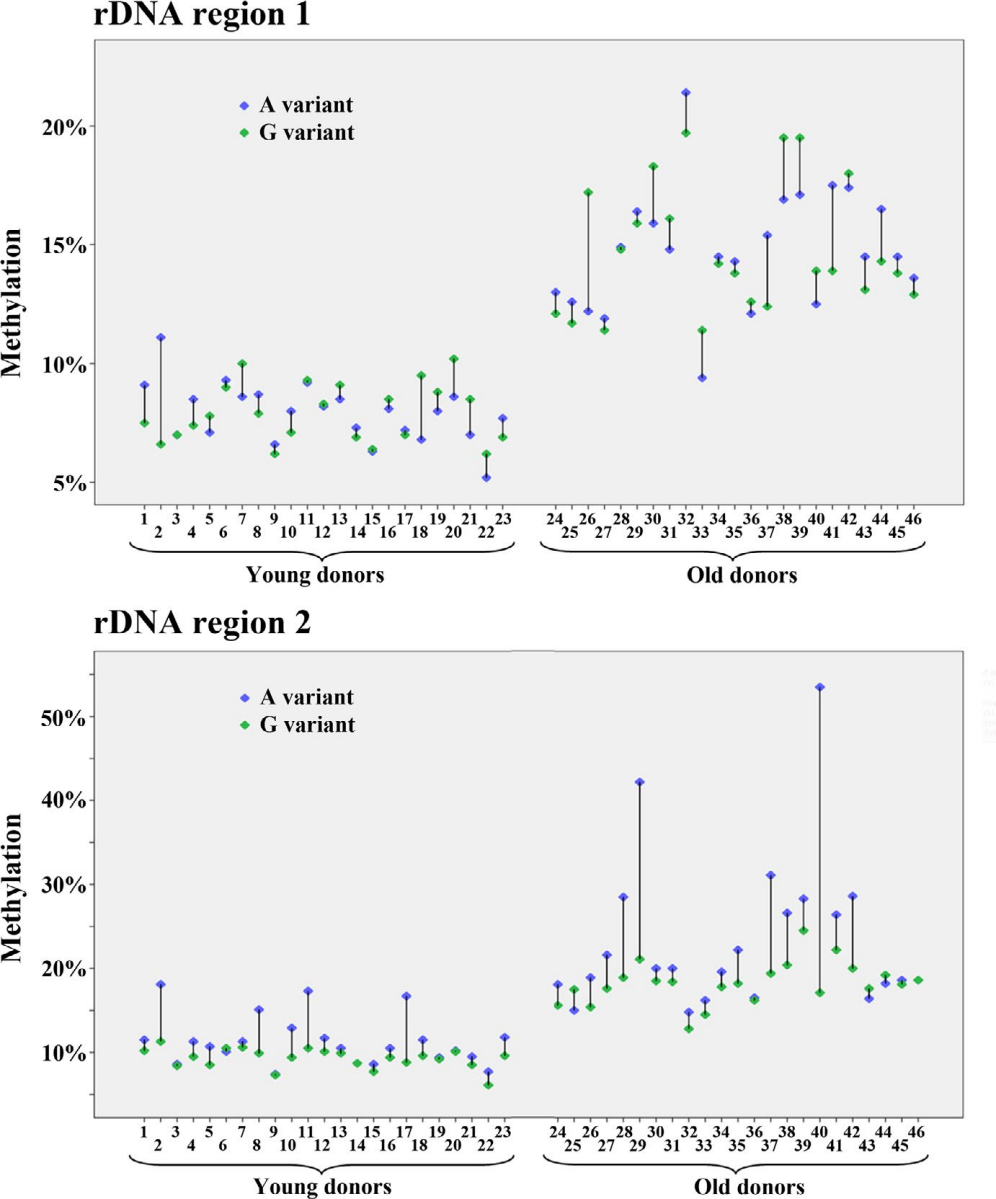
Allele‐specific methylation of rDNA region 1 (comprising 38 CpGs in the external transcribed spacer) and region 2 (25 CpGs in the upstream control element and core promoter) in sperm samples from young and old human donors. Twenty‐three informative samples from each age class were analyzed by deep bisulfite sequencing. Alleles with an A variant are indicated by a blue and alleles with a G variant by a green diamond symbol. Methylation of both alleles is increased in older males. Region 2 also shows a higher methylation of the A, compared to the G allele

The haploid sperm genome is endowed with several hundred copies of the rDNA transcription unit. Different alleles can be distinguished on the basis of single nucleotide polymorphisms (Babaian, 2017). We have developed deep bisulfite sequencing (DBS) assays for two regions of the rDNA transcription unit, each containing an A/G variant with a heterozygosity rate of around 35% (Table S4). Region 1 targets the external transcribed spacer (ETS) and region 2 the UCE and core promoter. For each variant, we selected 46 informative sperm samples, 23 from young donors (26–36 years) and 23 from old donors (43–60 years). DBS was used to determine variant‐specific methylation at single DNA molecule resolution. For both regions, we observed an increased average methylation level of both the A and the G allele in sperm samples of older donors (Figure 2; Table 2). Heat maps (Figure S2) showed an age‐dependent increase in methylation at each of the 38 analyzed CpGs in rDNA region 1 and the 25 CpGs in region 2. Interestingly, in region 2 the A allele was considerably higher methylated than the G allele in almost all analyzed sperm samples from both young and old donors (Figure 2). In region 1, there were comparable differences between allele‐specific methylation levels; however in a given sample, either the A or the G allele could be hypermethylated.

**TABLE 2:** Methylation and epimutation rates in sperm of young and old human donors, determined by deep bisulfite sequencing

	Donor's age class	Sample size (*N*)	Methylation (%) Mean ± *SD* [range]	Epimutation rate (%) Mean ± *SD* [range]
Region 1 A variant	Young	23	7.92 ± 1.24 [5.20–11.10]	0.32 ± 0.75 [0.00–3.58]
	Old	23	14.75 ± 2.53 [9.40–21.40]	1.36 ± 1.45 [0.00–4.78]
Region 1 G variant	Young	23	7.92 ± 1.22 [6.20–10.20]	0.23 ± 0.49 [0.00–1.78]
	Old	23	14.80 ± 2.70 [11.40–19.70]	1.00 ± 1.83 [0.00–6.94]
Region 2 A variant	Young	22	11.47 ± 2.95 [7.40–18.10]	1.61 ± 1.96 [0.00–6.13]
	Old	22	23.70 ± 9.38 [14.80–53.50]	9.52 ± 14.90 [0.16–62.98]
Region 2 G variant	Young	23	9.30 ± 1.19 [6.10–11.30]	0.42 ± 0.66 [0.02–3.02]
	Old	23	18.24 ± 2.52 [12.80–24.50]	2.30 ± 1.77 [0.14–5.88]

Abbreviation: *SD* = standard deviation.

Bisulfite pyrosequencing measures the average methylation of millions of DNA molecules in a genomic DNA sample. The observed methylation changes can be due to a gain of methylation at single CpG positions in a large number of different rDNA transcription units or to a few transcription units, where all or most CpGs become methylated. Unlike BPS, DBS can determine the methylation profiles of many thousand individual DNA molecules each in multiple samples in a single experiment. Because it is usually the density of CpG methylation in a cis‐regulatory region rather than individual CpGs that turns a gene “on” or “off,” most single CpG methylation errors represent stochastic aberrant methylation of one or a few CpGs in a target region with a much larger number of unmethylated CpGs and remain without functional consequences. In contrast, individual transcription units with the majority of CpGs being methylated are thought to be epigenetically silenced (Santoro & Grummt, 2001). In this light, allele methylation errors that are DBS reads with >50% methylated CpGs were considered as functionally relevant epimutations. Epimutation rates (ERs) were calculated by dividing the number of hypermethylated (>50%) alleles by the total number of reads.

For both variants and both analyzed regions of the rDNA transcription unit, sperm samples of the older donors displayed 4–6 times (*p* = 0.0001–0.005) higher ERs than those of younger males (Figure S3; Table 2). Sperm samples from old donors exhibited a higher range in rDNA methylation and ER variation than younger males (Figure 2 and Figure S3), indicating epigenetic drift in aging sperm. Apart from age, ERs also depended on sequence context. In both young and older donors, the ER of rDNA region 2 (UCE and core promoter) was 4–5 times (*p* = 0.001–0.002) higher than that of region 1 (ETS) (Figure S3). Consistent with the overall higher methylation of the A alleles, compared to the G alleles in region 2, we also observed a 4 times higher ER of A alleles (Table 2).

### 2.3 rDNA methylation in cord blood and peripheral blood

We have shown previously that paternal age can influence average methylation of particular genes in fetal cord blood, which represents a mixture of paternal and maternal alleles (Atsem et al., 2016). To study the possible transmission of sperm epigenetic signatures to the next generation, we performed BPS of the rDNA UCE and promoter in cord bloods of 121 children conceived by IVF/ICSI with sperm samples of cohort 2 (Figure S4). We observed a slight increase of rDNA methylation with age of the father at conception; however, the results were not significant (Pearson's *r* = 0.01; *p* = 0.92 for the UCE and *r* = 0.06; *p* = 0.53 for the promoter).

**FIGURE 3:**
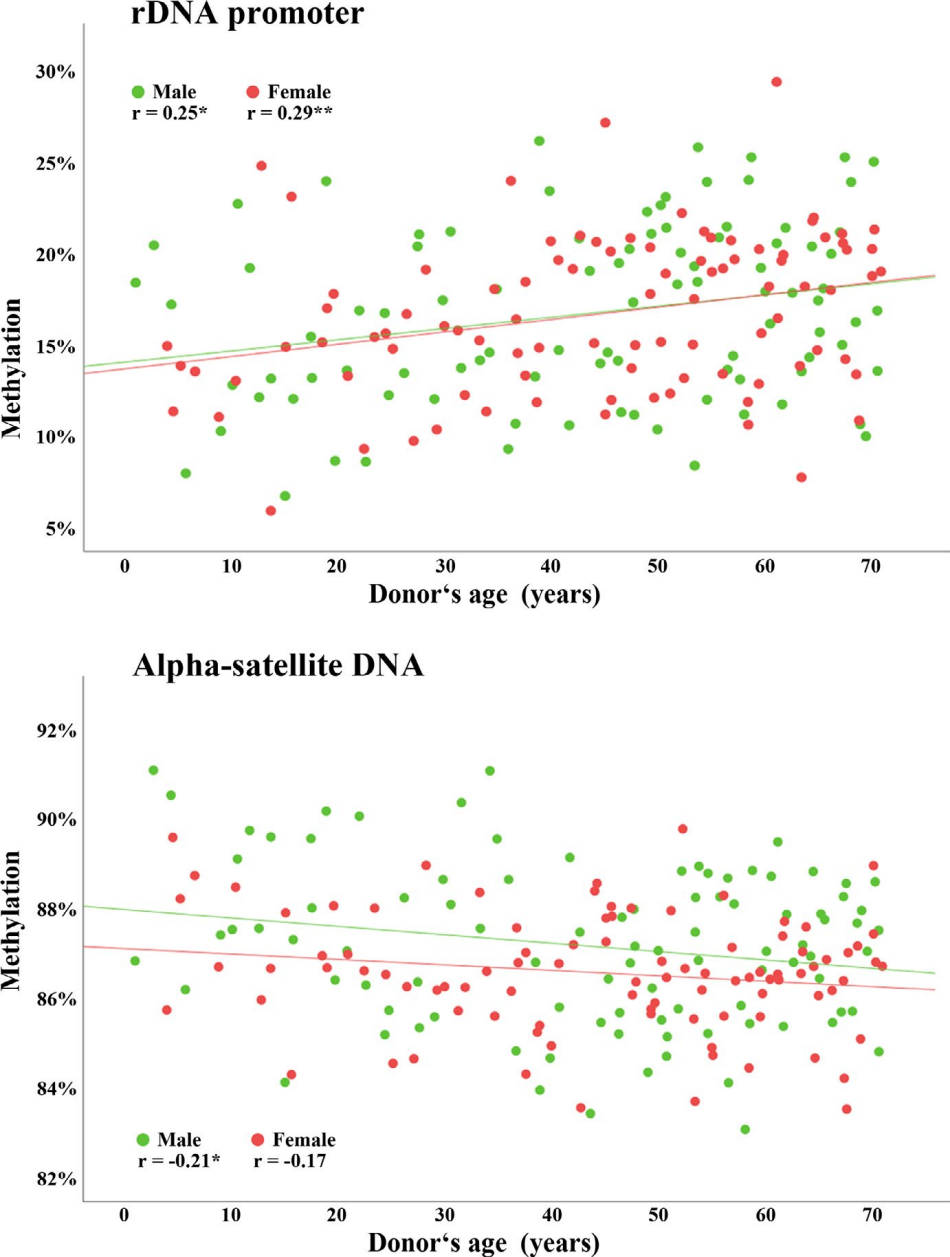
Methylation of the rDNA core promoter and α‐satellite DNA in human peripheral blood, determined by bisulfite pyrosequencing. rDNA (9 CpGs) methylation increases, whereas α‐satellite DNA (4 CpGs) methylation decreases with donor's age. Scatter plots show significant (*p < 0.05 and **p < 0.01) correlations in 94 samples each from males (green dots) and females (red dots). Pearson's correlations were used for statistical analysis

The effect of paternal age on offspring's blood methylation, if any, is concealed by a much larger effect of aging of the individual. To demonstrate this, we determined the methylation levels of rDNA promoter and α‐satellite DNA in peripheral blood samples of 94 males and 94 females, ranging from 1 to 70 years in age. There was a highly significant positive correlation (Pearson's *r* = 0.27; *p* < 0.0001) between blood rDNA methylation and donor's age across all 188 samples. Similar age effects were seen in male (Pearson's *r* = 0.25; *p* = 0.016) and female (*r* = 0.29; *p* = 0.004) samples (Figure 3). Consistent with earlier studies (Flunkert et al., 2018), blood α‐satellite DNA methylation decreased with age (Pearson's *r* = −0.21; *p* = 0.046 in males and *r* = −0.17; *p* = 0.098 in females).

### 2.4 Sperm rDNA clock

**FIGURE 4:**
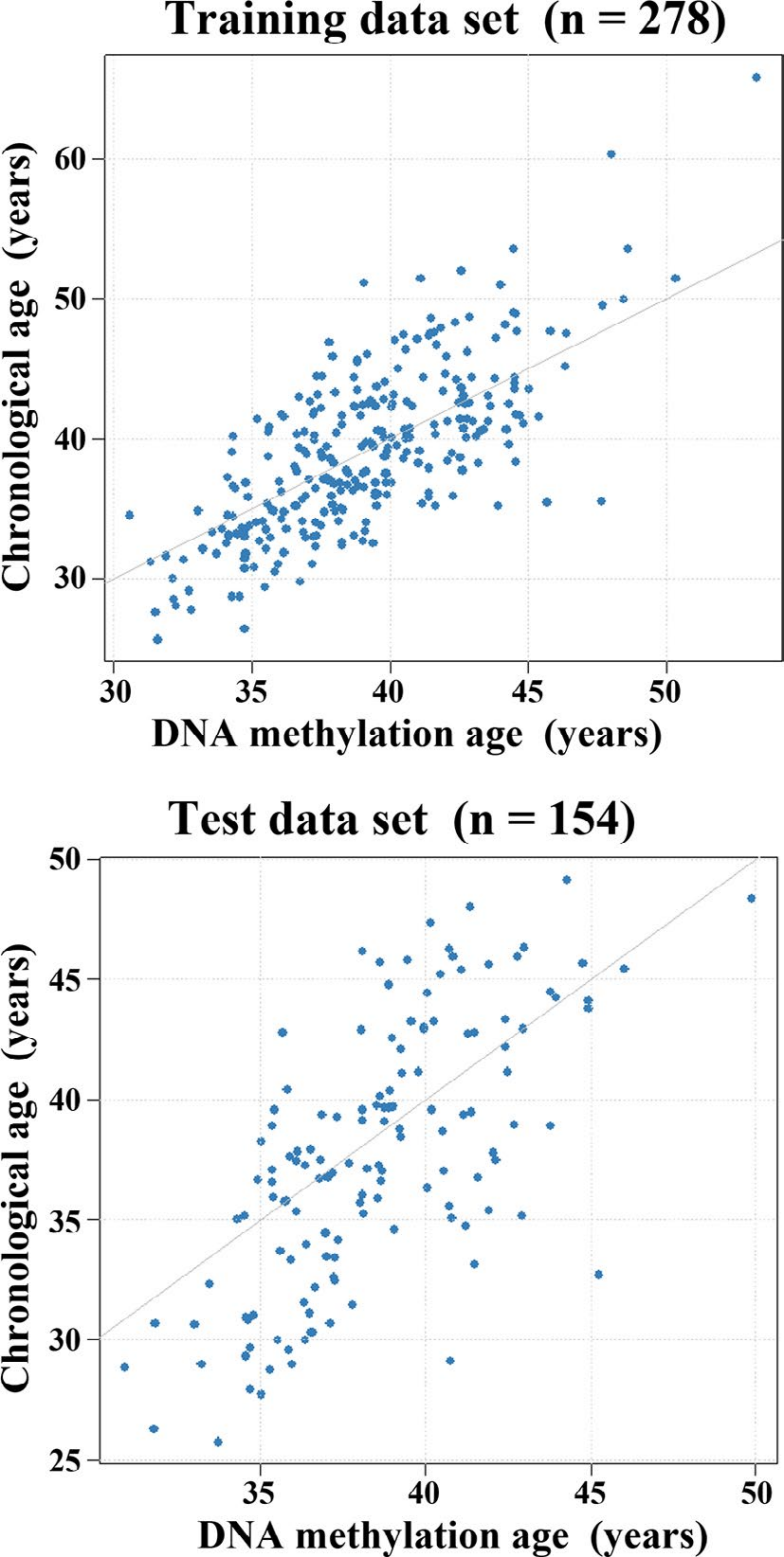
Building the rDNA methylation clock. Scatter plots showing the chronological age (*y*‐axis in years) versus rDNA methylation age (*x*‐axis in years) in the training (*N* = 278) and testing cohort (*N* = 154). Model performance on training cohort: mean squared error (*MSE*) = 16.30, median absolute difference (MAD) = 2.78, Pearson's *r* = 0.72; and on test cohort: *MSE* = 16.96, MAD = 2.91, *r* = 0.67

Specific CpGs in the rDNA transcription unit can be used to predict the chronological age of the donor from sperm methylation. As detailed in the methods section, we fitted an ElasticNet regression model using methylation values of the 53 analyzed CpG sites in four different BPS amplicons. The formula estimates the methylation age from the ElasticNet regression model based on rDNA methylation data. The estimated age is essentially a weighted average of the methylation β values measured at distinct CpG sites including a baseline (intercept) offset. Using tenfold cross‐validation, a model with 15 CpGs including three in the rDNA core promoter, 10 in the UCE, and two in 28S rDNA, but none in 18S rDNA, was selected. In the training data set with 278 samples from sperm cohorts 1 and 2, we observed a Pearson's correlation coefficient between DNA methylation age (predicted age) and chronological age of 0.72 and a median absolute difference (MAD) between predicted and chronological age of 2.78 (Figure 4). In an independent test data set with 154 samples from cohort 3, the predictive error was only slightly lower (*r* = 0.67, MAD = 2.91), meaning that in over half of the cases the methylation age differs less than three years from the chronological age (Figure S5).

### 2.5 Evolutionary conservation of age‐related sperm rDNA methylation

**FIGURE 5:**
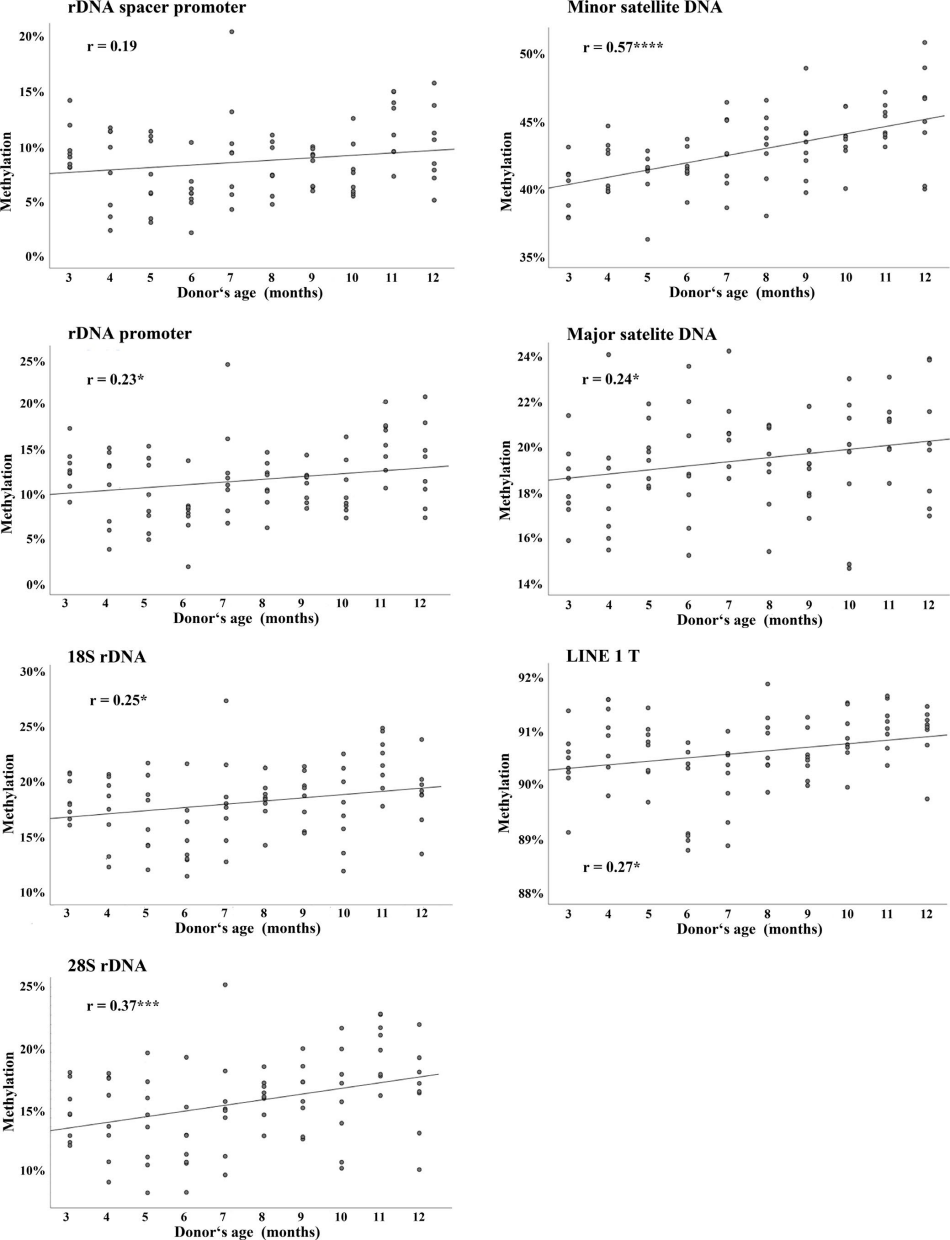
Repeat methylation in mouse sperm increases with donor's age. Scatter plots show significant (*p < 0.05, ***p < 0.001, and ****p < 0.0001) Pearson's correlations between donor's age (*x*‐axis in years) and mean methylation (*y*‐axis in %) of the rDNA spacer promoter (14 CpGs), rDNA gene promoter (7 CpGs), 18S rDNA (8 CpGs), 28S rDNA (10 CpGs), minor satellite DNA (2 CpGs), major satellite DNA (3 CpGs), and LINE1‐T repeats (4 CpGs). Eighty sperm samples from 3‐ to 12‐month‐old mice were analyzed by bisulfite pyrosequencing

To uncover whether the paternal age effect is evolutionarily conserved, sperm DNA methylation was analyzed in mouse, bovine, and marmoset, a small new‐world primate. Eighty sperm samples were collected from 3‐ to 12‐month‐old mice (8 samples each per month). At most two animals were taken from the same litter. There was a significant increase in methylation of rDNA (spacer and gene promoter, 18S and 28S), (peri)centromeric minor and major satellite DNA, and interspersed LINE1 T repeats with donor's age (Figure 5; Table S5).

**FIGURE 6:**
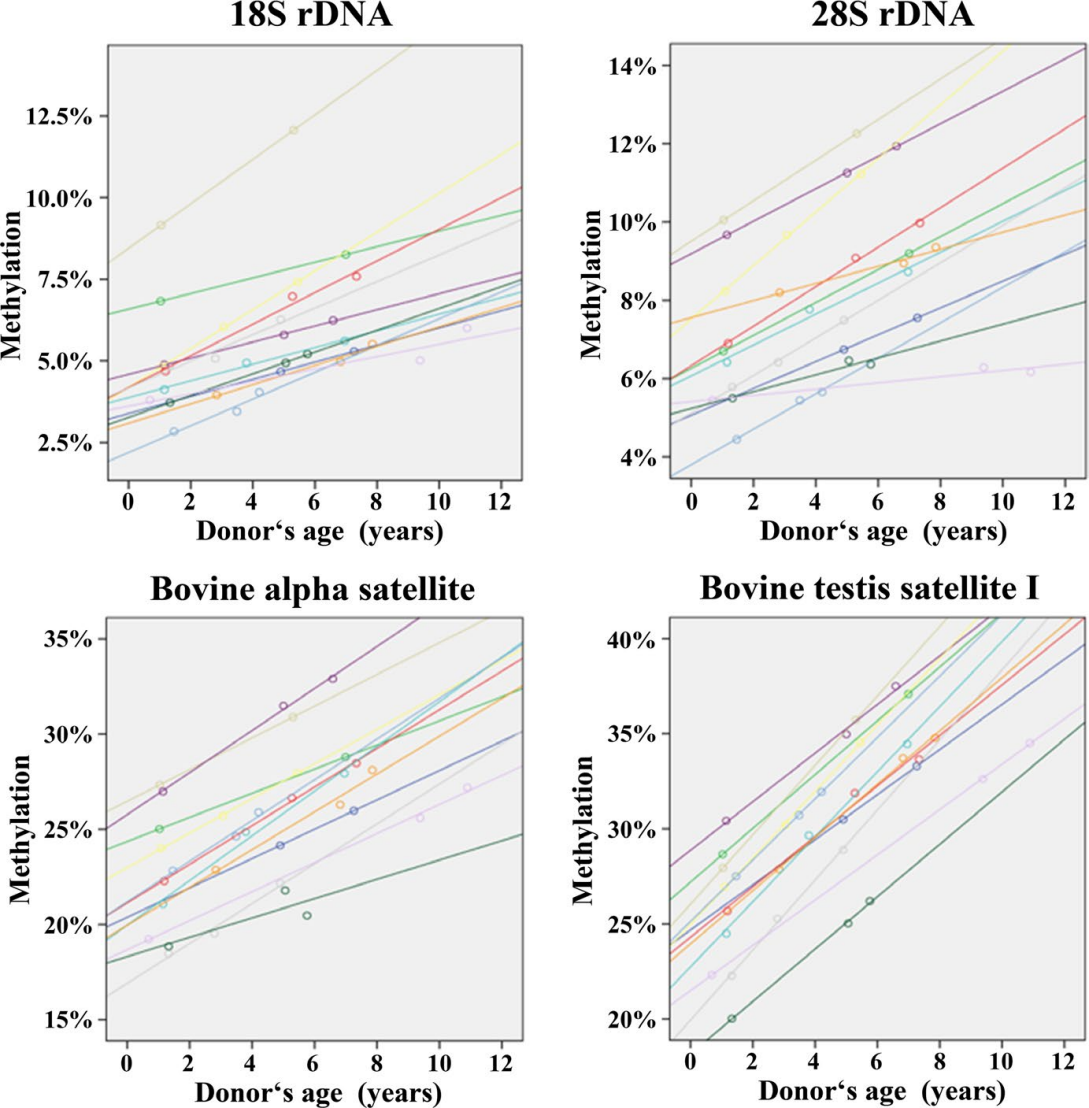
Sperm methylation of 18S rDNA (8 CpGs), 28S rDNA (10 CpGs), bovine alpha‐satellite (12 CpGs), and bovine testis satellite I (9 CpGs) increases with donor's age. The color‐coded regression lines show the age‐related increase in DNA methylation of sperm samples from 12 bulls, analyzed by BPS at 2–3 different ages

Similarly, 36 sperm samples from 15 different bulls were analyzed by BPS. From nine bulls, we had samples at young (<3 years), middle (3–6 years), and old age (>6 years), and from three bulls at two different ages. Both 18S and 28S rDNA as well as bovine alpha‐ and testis satellite I DNA showed a significant correlation between methylation and donor's age (Table S5). For each of the 12 bulls with samples at different ages and each amplicon analyzed, DNA methylation increased from young, to middle, to old age (Figure 6).

We also analyzed 16 sperm samples from 1‐ to 12‐year‐old marmosets. There was a slight increase in 18S and 28S rDNA methylation with donor's age but due to the small sample size, the correlations were not significant. There was a highly significant positive correlation between centromeric α‐satellite DNA methylation and donor's age (Figure S6, Table S5).

Since the primers for 18S and 28S rDNA are conserved across all analyzed species, it was possible to compare the methylation changes of orthologous regions in the rDNA transcription unit. To this end, methylation of a given sample was adjusted to the life span, which is largely different between mouse (28 months), bull (20 years), marmoset (12 years), and humans (80 years). Although average sperm rDNA methylation and methylation increase during life span differed to some extent between species (Table S6), combined analysis of 80 murine, 36 bovine, 16 marmoset, and 295 human sperm samples was consistent with an evolutionarily conserved age effect on 18S and 28S rDNA methylation (Figure S7).

## 3 DISCUSSION

### 3.1 Age‐dependent methylation of rDNA and other repetitive elements in sperm

More than half of the CpG sites in the human genome reside in repetitive DNA elements. The DNA methylation levels differ between various repeat types and in response to a wide range of external and internal factors. Methylation of ALU and LINE1 elements prevents retrotransposon activity and genome instability (Yoder, Walsh, & Bestor, 1997). Retrotransposons can modulate the local epigenetic landscape through changes in DNA methylation and, thus, confer phenotypic plasticity upon environmental exposures (Sharif, Shinkai, & Koseki, 2013).

Sperm methylation of rDNA, α‐satellite DNA, and LINE1 methylation significantly increased with donor's age. Our results are consistent with an earlier study demonstrating LINE1 hypermethylation in aging sperm (Jenkins et al., 2014). One explanation for the susceptibility of various repeat classes to paternal age is chromatin remodeling during spermiogenesis. Although most DNA in mature sperm is packaged by protamines into an almost crystalline structure, rDNA, centromere repeats, LINE, and SINE retrotransposons appear to preserve nucleosomes and a more open chromatin configuration (Samans et al., 2014; Sillaste et al., 2017). Paternally derived nucleosomes are generally retained at gene promoters and genomic loci that play an important role during embryogenesis (Hammoud et al., 2009; Samans et al., 2014), including many genes for RNA processing factors.

Previously, we have shown that rDNA methylation increased, whereas LINE1 and α‐satellite DNA methylation decreased during in vitro aging of human fibroblasts (Flunkert et al., 2018). This is also true for blood samples from aging individuals. Thus, the rDNA behaves similarly in sperm and somatic cells/tissues, whereas α‐satellite DNA and LINE1 show opposite correlations in germline and soma with age. This argues in favor of the notion that repeat methylation is not a mere side effect of aging process, but the product of specific mechanisms, which can distinguish different repeat classes in germ cells and soma.

### 3.2 Mechanisms underlying age‐associated hypermethylation of sperm repetitive DNA

Male germline‐specific DNA methylation patterns are established after prenatal mitotic arrest of spermatogonia and completed postnatally during meiosis (Boyano, Andollo, Zalduendo, & Arechaga, 2008). By immunolocalization, it was shown that the DNA methyltransferases DNMT1, DNMT3A, and DNMT3B are present in spermatogonia and spermatocytes. *DNMT1* and *DNMT3A* mRNAs are also detected in mature sperm (Marques et al., 2011). In somatic cells, methylation of rDNA critically depends on DNMT1 and DNMT3B activity (Schmitz, Mayer, Postepska, & Grummt, 2010), that of LINE1 on DNMT3B (Choi et al., 2011), that of α‐satellite DNA on DNMT3B and to a lesser extent on DNMT3A (Choi et al., 2011). In the germline, de novo methylation of interspersed repeats also requires the catalytically inactive DNMT3L. The demethylating enzymes TET2, TET1, and TET3 are expressed successively in spermatocytes and spermatids. Decreased *TET1‐3* mRNAs are found in sperm with low concentration and fertilization rates (Ni et al., 2016). Increased DNMT (most likely DNMT3B and DNMT3L) and/or decreased TET activities during spermatogenesis of old males could explain the age‐related gain of repeat methylation in sperm.

We observed increasing sperm methylation with age for all analyzed CpGs (53 by BPS and 63 by DBS) covering different regions (ETS, UCE, core promoter, 18S rDNA, and 28S rDNA) of the human rDNA transcription unit. Single‐molecule sequencing suggests that the gain in methylation is at least partially due to an increasing rate of hypermethylated transcription units, which negatively influence rRNA expression (Santoro & Grummt, 2001). Methylation of the upstream non‐transcribed spacer may facilitate 5′ to 3′ methylation spreading during aging or age‐related diseases (Turker, 2002).

### 3.3 Functional implications of age‐related sperm repeat DNA methylation for the next generation

The rDNA transcription unit contains more than 1,500 CpG sites whose methylation is involved in epigenetic rDNA silencing (Santoro & Grummt, 2001; Wang & Lemos, 2019). Our results demonstrate that sperm rDNA is subject to the same age‐related increase in DNA methylation as somatic tissues. In somatic cells, rDNA methylation reflects changes in nucleolar biology during aging and in age‐related conditions (Wang et al., 2015). Therefore, rDNA methylation is a primary candidate when looking for age‐related sperm epigenetic signatures which might have an impact on the next generation. By BPS, we did not observe a correlation between paternal age and rDNA methylation in cord blood of children. Although detection of a paternal age effect is hampered by the presence of maternal rDNA, we can largely exclude effect sizes similar to those observed in sperm. On the other hand, the most obvious consequences of increased sperm rDNA methylation can be expected shortly after fertilization, when restoration of totipotency and epigenetic genome reprogramming occur (Reik et al., 2001).

Activation of RNA polymerase I, rDNA synthesis, and formation of the nucleolus mark activation of the embryonic genome, which occurs at the two‐cell stage in mouse and at the eight‐cell stage in humans (Eckersley‐Maslin, Alda‐Catalinas, & Reik, 2018). The early embryo is the fastest dividing tissue and, therefore, highly dependent on efficient ribosome biogenesis and protein synthesis. The nucleolus is the most prominent and, arguably, the most important cellular machinery in the early embryo. A higher methylation and, by extrapolation, lower transcription of the paternal rDNA component during early embryogenesis may have immediate functional consequences and primarily affect embryo metabolism. We propose that increased rDNA methylation in sperm of old males decreases the developmental potential of the resulting embryos, contributing to age‐related fertility problems. Disorganization or delayed formation of the nucleolus is an indicator of aberrant nuclear reprogramming in cloned embryos (Fléchon, 2006).

Most LINE1 elements in the human genome are truncated; only about 100 full‐length copies are still active. Intragenic LINE1 elements can act as cis‐regulatory elements. LINE1 expression and retrotransposition occur in the germline, during embryogenesis, and to some extent in somatic tissues (Kano et al., 2009). The decreasing LINE1 methylation with age is thought to slowly erode genome integrity in the soma, contributing to biological aging (St. Laurent, Hammell, & McCaffrey, 2010). Sperm LINE1 methylation (mean 70%, range 20%–80%) was comparable to that of somatic cells. Notably, approximately 5% of sperm samples displayed low (20%–50%) LINE1 methylation, which may represent a threat to genome stability. In this respect, an age‐dependent increase in sperm LINE1 methylation may be advantageous. Due to drastic demethylation of the paternal genome after fertilization (Reik et al., 2001), the highest level of LINE1 activity is observed in the mouse 2‐cell embryo, which then decreases up to the 8‐cell stage. Intuitively, this could be considered as an undesirable side effect of epigenetic reprogramming. However, accumulating evidence (Jachowicz et al., 2017; Peaston et al., 2004) suggests that expression of specific classes of repetitive elements in early embryogenesis regulates global chromatin accessibility and is essential for the highly coordinated activation of developmental programs. The increasing methylation of sperm LINE1 elements with paternal age could interfere with their timely reactivation after fertilization, decreasing developmental rates (Jachowicz et al., 2017).

In vitro aging and irradiation of human fibroblasts are associated with decreasing α‐satellite methylation (Flunkert et al., 2018). Reduced methylation of centromeric satellite DNAs may contribute to chromosomal instability in cancer cells (Bollati et al., 2009). In contrast to somatic cells/tissues, sperm aging is associated with a gain of α‐satellite methylation. However, in this context, it is worth emphasizing that sperm α‐satellite shows considerable undermethylation (mean 35%), compared to somatic tissues such as embryonal fibroblasts (78%–85%; Flunkert et al., 2018) and blood (83%–91%). If hypomethylation of centromeric satellite DNAs is an epigenetic mark to discriminate germ cells from somatic cells (Yamagata et al., 2007), increasing methylation levels may affect the reprogramming of the sperm epigenome after fertilization.

### 3.4 Human sperm rDNA clock

To date, two epigenetic clocks for human sperm DNA have been developed, based on the analysis of Illumina 450K methylation array data. Lee et al. (2015) presented an age‐predictive linear regression model trained on 3 CpGs. This comparatively small model achieved a MAD of 5.4 years on a validation cohort. Jenkins, Aston, Cairns, Smith, and Carrell (2018) focused on a pre‐selected set of CpGs aggregated in 51 regions. They used a similar approach (ElasticNet regression) as ours to build a model, yielding a mean absolute error of 2.37 years on an independent cohort, compared to 2.04 years in the training data set. Because clock CpGs were selected from a much larger number of array CpGs, their model gives higher correlations (*r* ^2^ = 0.76 in the test and 0.89 in the training cohort) than ours (*r* ^2^ = 0.45 and 0.52, respectively), which is based on 15 of 53 CpGs in four rDNA amplicons.

While the role of methylation changes in the highly selected array CpGs for biological aging processes remains largely unclear, our sperm rDNA clock may reflect changes in nucleolar activity which are functionally related to biological aging (Wang et al., 2015). Wang and Lemos (2019) suggested that rDNA methylation can serve as a universal predictor of age in different somatic tissues and species. They developed rDNA models for mouse (and other species) based on reduced representation and whole‐genome bisulfite sequencing data. A mouse model built exclusively on mouse–human homologous CpGs was applied to skin samples of 6 human adults and yielded a strong positive correlation between rDNA methylation age and chronological age. A systematic evaluation of the model was not performed, precluding a direct comparison to our model, but their data corroborate the conservation of the age‐related methylation. Here, we demonstrate an rDNA clock which works in human sperm. Both sperm, which has undergone germline reprogramming, and peripheral blood, which represents somatic tissue, showed an increase of rDNA methylation with donor's age. Notably, the paternal age effect on sperm (0.33% increments per year) was much larger than that on blood (0.06%). One important advantage of our BPS‐based rDNA clock is that reliable measurements can be obtained with very low amounts of DNA equivalent to 10 sperm cells. This is essential for forensic analyses of semen traces, when array or NGS‐based methylation tests fail.

## 4 CONCLUSIONS

Paternal age is associated with increased sperm methylation of rDNA and other repetitive DNA families, which preserve nucleosomes and a more open chromatin organization in mature sperm. The paternal age effect on sperm rDNA and repeat methylation has been conserved in different mammalian species, which is generally considered as a good indicator of functional significance. We propose that increased methylation of paternal rDNA transcription units may interfere with nucleolar structure and function in the early embryo and, directly or indirectly, with developmental potential.

We have developed a human sperm rDNA clock which works with very low amounts of DNA, however, for clinical applications the performance must be improved, that is, by the analysis of all 1,500 CpGs in the rDNA transcription unit. Although the correlations described here are relatively low, increasing methylation with paternal age was observed for all analyzed CpGs (by two different techniques) in humans and other species. One important point is considerable sperm rDNA methylation variation (10%–15%) among individuals of comparable age within the same species. This biological variation may be due to genetic variants, stochastic factors during germline reprogramming, environmental exposures, and lifestyle. Technical variation is in the order of only 1%–2%. Overall, the effect size of paternal age may be small, compared to other factors, and/or paternal age may be only one of many factors shaping the sperm epigenome.

The DNA methylation age of germ cells can deviate considerably from chronological age, which is at least partially due to biological variation. Preliminary evidence suggests that somatic DNA methylation age‐based predictors of human morbidity and mortality can be reversed by pharmacological aging intervention (Fahy et al., 2019). It is interesting to speculate whether sperm with low rDNA methylation show a higher developmental potential of the resulting embryos than sperm with high DNA methylation age. If so, it may be possible to decrease a given donor's sperm methylation and increase his fertility by certain diets or drugs.

## 5 MATERIALS AND METHODS

### 5.1 Study samples

The study on human sperm and blood samples was approved by the ethics committee at the medical faculty of the University of Würzburg (no. 117/11 and 212/15). After in vitro fertilization (IVF) or intracytoplasmic sperm injection (ICSI) at the Fertility Center Wiesbaden, the left‐over swim‐up sperm fraction (excess material) was collected, pseudonymized, and frozen at −80°C. After thawing, the swim‐up sperm samples were purified further by density gradients PureSperm 80 and 40 (Nidacon, Mölndal, Sweden). Fetal cord bloods from newborn singletons conceived through IVF/ICSI were collected by collaborating obstetric clinics throughout Germany. For sperm cohort 1 (186 samples, Table S1), the outcome of fertility treatment was unknown. For sperm cohort 2 (109 samples, Table S1), cord bloods of the resulting children were available. Peripheral blood DNAs from 94 male and 94 female mutation‐negative individuals with an age range from 1 to 71 years were anonymized excess materials from predictive genetic diagnostics.

Mouse (*Mus musculus*) sperm samples were isolated from 3‐ to 12‐month‐old mice after cervical dislocation. The vas deferens and caudal epididymis were dissected and placed separately into 500 µl GMOPS with 10 mg/ml human serum albumin at 37°C. After repeated washing, the final fraction was resuspended in 500 µl 1xPBS and stored at −80°C.

Sperm samples of 15 high‐performance breeding bulls (*Bos taurus*) were obtained from Masterrind, Verden, Germany. Two or three samples were available from 12 bulls at different ages. Bull sperm samples were purified by BoviPure and BoviDilute (Nidacon) following the manufacturer's protocol.

Sperm samples from common marmosets (*Callithrix jacchus*) were obtained by penile vibrostimulation of male animals housed at the German Primate Center in Göttingen. Swim‐up purification of sperm was performed after density gradient purification of fresh sperm samples. Animal experiments were approved by the Niedersächsisches Landesamt für Verbraucherschutz und Lebensmittelsicherheit (no. 42502‐04‐17/2496).

For DNA isolation, the purified sperm cells were resuspended in 300 µl buffer (5 ml of 5 M NaCl, 5 ml of 1 M Tris‐HCl; pH 8, 5 ml of 10% SDS; pH 7.2, 1 ml of 0.5 M EDTA; pH 8, 1 ml of 100% β‐mercaptoethanol, and 33 ml H_2_O) and 100 µl (20 mg/ml; 600 mAU/ml) proteinase K (Qiagen, Hilden, Germany), and incubated for 2 hr at 56°C. Sperm DNA was isolated using the DNeasy Blood and Tissue kit (Qiagen), and blood DNA by the classical salting‐out method. DNA concentration and purity were measured by NanoDrop 2000c spectrophotometer (Thermo Scientific, MA, USA). Bisulfite conversion of DNA was performed using the EpiTect Fast 96 Bisulfite Kit (Qiagen).

### 5.2 Bisulfite pyrosequencing

PCR and sequencing primers (Table S2) for human rDNA (UCE, core promoter, 18S rDNA, and 28S rDNA), α‐satellite DNA, ALU, and LINE1, marmoset α‐satellite DNA, mouse rDNA (spacer and gene promoter), (peri)centromeric minor and major satellite DNA, and interspersed LINE1‐T, bovine alpha‐satellite and testis satellite I DNA were designed using the PyroMark Assay Design 2.0 software (Qiagen).

Fully methylated and unmethylated DNA standards (Qiagen) were used as controls in each run. PCRs were carried out using ~25 ng bisulfite‐converted DNA and FastStart Taq DNA polymerase (Roche Diagnostics, Mannheim, Germany). Amplifications were performed with an initial denaturation at 95°C for 5 min, 35 cycles of 95°C for 30 s, primer‐specific annealing temperature (Table S2) for 30 s, and 72°C for 45 s, and a final extension step at 72°C for 10 min. Pyro Q‐CpG software (Qiagen) and PyroMark Gold Q96 CDT reagent kit were used to perform pyrosequencing on the PyroMark Q96 MD system.

### 5.3 Deep bisulfite sequencing

DBS primers (Table S4) were designed for human rDNA region 1 (ETS) and region 2 (UCE and core promoter). First‐round PCRs were carried out using ~50 ng bisulfite‐converted DNA and FastStart Taq DNA polymerase. Artificially 0%, 50%, and 100% methylated DNAs (Qiagen) served as controls. PCR products were cleaned with Agencourt AMPure XP Beads (Beckmann Coulter, Krefeld, Germany), quantified using Qubit dsDNA BR Assay system kit (Invitrogen, Karlsruhe, Germany), and diluted to a concentration of 0.2 ng/µl. In a second PCR, the samples from different assays were pooled together and barcoded using 48 multiplex identifiers. NEBNext Multiplex Oligos (Dual Index Primer Set 1) for Illumina (New England BioLabs, Frankfurt, Germany) were used for adaptor ligation. The purified and quantified PCR pools were diluted to a concentration of 4 nM, and 3 µl of this dilution from each of the 48 MIDs were pooled together into one final pool for next‐generation sequencing.

Paired‐end (250 bp) sequencing was performed using the Illumina MiSeq and Reagent Kit v2 (500 cycles) cartridge (Illumina, CA, USA) according to the manufacturer's instructions. After the run, the sequencing reads were processed by Illumina Genome Analyzer. FASTQ files were analyzed further using the Amplikyzer2 software (https://bitbucket.org/svenrahmann/amplikyzer/wiki/Installation) and in‐house R scripts. To determine allele‐specific methylation rates, all analyzed amplicon sequences were aligned to the reference genomic sequence for each assay, and allele splitting was done based on the genetic variant within the target region. Only reads with an overall bisulfite conversion rate of >95% were considered.

### 5.4 Statistical analyses

Statistical analysis was performed using the software R (version 3.2.2) and the packages of the Bioconductor project (https://www.bioconductor.org/). Male donor's age was correlated with the sperm DNA methylation level of the corresponding amplicon. For human samples, Pearson's partial correlations were applied to adjust for possible confounding factors. For animal models, either Pearson's or Spearman's correlations were used depending on data distribution. Parametric *t* tests or non‐parametric Mann–Whitney *U* tests were performed for group comparisons.

Building of the rDNA clock model was performed with a training cohort of 289 samples and validated on an independent test cohort of 154 samples. The β values of 53 CpG sites in the four analyzed rDNA gene regions were used as features. Samples with 10 or more missing values in the CpG measurements were removed, yielding a final training cohort of 278 samples. For the remaining samples, missing CpG values were imputed using the K‐nearest neighbor approach of the impute.knn function in the impute package (https://www.bioconductor.org/packages/3.2/bioc/html/impute.html) with default settings. An ElasticNet model was fitted as implemented in the glmnet package (https://cran.r‐project.org/web/packages/glmnet/index.html) with default parameter settings. The parameter α which selects between lasso and ridge penalty was set to α = 0.5 as in previous studies (Horvath, 2013). Selection of the penalizing parameter lambda was performed based on tenfold cross‐validation on the training set, using the mean square error (*MSE*) loss function. A lambda yielding an *MSE* of one standard error above the minimal *MSE* was chosen for the final model (λ = 1.15). This model contained 15 features in total. To obtain an estimate of the prediction error, the final model was validated on an independent cohort.

## CONFLICT OF INTEREST

None declared.

## AUTHOR CONTRIBUTIONS

T.Hf. designed the study. T.Hf. and R.P. wrote and revised the manuscript. R.P. performed all the experiments and analyzed the data. N.E.H. supervised the experiments. M.D. and T.M. designed the epigenetic clock. M.S. and T.Hn. contributed human study samples. F.Z. and G.E.P. contributed mouse study samples. A.L‐H. and H.N. contributed bovine study samples. C.D. and R.B. contributed marmoset study samples. All authors reviewed and approved the final manuscript.

## Supporting information

Supplementary Material

## Data Availability

The DBS data set is deposited in NCBI's Sequence Read Archive (number SUB6706443). The script for the sperm rDNA clock will be made available upon request.
