# Supplementary material for: Increasing methylation of sperm rDNA and other repetitive elements in the aging male mammalian germline
Source: Aging Cell. 2020 Jul 1;19(8):e13181. doi: 10.1111/acel.13181 (PMC7431825; doi:10.1111/acel.13181)
Supplement: Supplementary file 1 — Supplementary Material [file ACEL-19-e13181-s001.pdf]

TABLE S1 Clinical parameters of the analyzed human sperm samples

**Parameters**	**Cohort 1** Mean ± SD* [range]	**Cohort 2** Mean ± SD* [range]	**Cohort 3** Mean ± SD* [range]
Sample size (N)	186	109	154
Smokers (N)	11	14	16
Donor’s age (years)	39.0±6.4 [25.7-65.8]	38.9± 5.1 [28.1-52.9]	37.8±5.4 [25.8-49.2]
Body mass index (kg/m^2)	25.7±2.9 [19.4-36.8]	25.8±3.4 [17.3-40.3]	26.3±3.8 [17.5-38.7]
Sperm concentration (million/ml)	28.6±33.4 [0.2-210]	38.1±40.6 [0.2-200]	43.2±51.6 [0.5-250]

* SD = standard deviation.

TABLE S2 PCR and pyrosequencing primers for human, mouse, bovine, and marmoset repetitive elements

Assay	Primer	Sequence (5'-3')[a]	Number of CpGs	Annealing temperature
Human rDNA upstream control element[c]	Forward outer[b]	GTGTTTTGGGGTTGATTAGAGG		60°C
	Reverse outer[b]	ATCACCRATAAACCAAAACCCC		
	Forward	GTGTTTTTGGGTTGATTAGAGG		
	Reverse	*CATCCCAAAACCCAACCTCTCC		
	Sequencing 1	GGTTGATTAGAGGGTT	5	
	Sequencing 2	TTTTGGGGATAGGTG	13	
	Sequencing 3	TTYGGGGGAGGTATATTTT	8	
Human rDNA promoter[d]	Forward outer[b]	GGTAGTTGTYGAGGGAGGGG		60°C
	Reverse outer[b]	AAAAAAACRTCCCCAACCTCC		
	Forward	GTTTTYGTTGTGAGTTAGGTAGAGTTT		60°C
	Reverse	*AAAAAAACRTCCCCAACCTCC		
	Sequencing	GGTTTATGTGGGGGAGAGGTTGT	9	
Human, mouse, bovine, and marmoset 18S rDNA	Forward	AGGTTTGTGATGTTTTTAGATGT		60°C
	Reverse	*AAAACCTCACTAAACCATC		
	Sequencing 1	TTTGTGATGTTTTTAGATGTT	4	
	Sequencing 2	ATTAAGTTTTTGTTTTTTGTATATA	4	
Human, mouse, bovine, and marmoset 28S rDNA	Forward	GGTTTTAAGTAGGAGGTGTTAGAAAAG		60°C
	Reverse	*CAACCAAACACATACACCAAATATCT		
	Sequencing 1	GGATAATTGGTTTGTGG	7	
	Sequencing 2	GTTGGATTGTTTATTTATTAATAGG	3	

Human α-satellite DNA	Forward	*TGTAAGTGGATATTTGGATTATTGG		57°C
	Reverse	AACAATTTCAAAACTACTCCATCAA		
	Sequencing	CTCAAAAATTTCTAAAAATACTTCTC	4	
Human LINE1	Forward	TTTTGAGTTAGGTGTGGGATA		60°C
	Reverse	*CTCACTAAAAAATACCAAACAA		
	Sequencing	GTTAGGTGTGGGATATAGTT	4	
Human ALU[e]	Forward	GGGACACCGCTGATCGTATATTTTTATTAAAAATATAAA		52°C
	Reverse	CCAAACTAAAATACAATAA		
	Universal	*GGGACACCGCTGATCGTATA		
	Sequencing	AATAACTAAAATTACAAAC	3	
Mouse rDNA spacer promoter[f]	Forward	GGAGAAGTGGTGGGTGGG		60°C
	Reverse	*CTCCTATATCACCAACCTAAAAAACCT		
	Sequencing 1	AGTGGTGGGTGGGTA	5	
	Sequencing 2	AGTGAGTGAATGTGG	4	
	Sequencing 3	ATTGGTTTGTATGGTTGA	3	
	Sequencing 4	GATATTTAGTGGTGATAAGTTT	2	
Mouse rDNA core promoter[f]	Forward	TTGGGGAGGTGGTTTAAAAATGA		60°C
	Reverse	*CCTCCAAAAACCCTCTCTAT		
	Sequencing 1	GAGGTGGTTTAAAAATGAT	2	
	Sequencing 2	GGATTTTAAAGGAATAATTGGT	3	
	Sequencing 3	GATAGGTTAATGAAAGAAAA	2	
Mouse minor satellite DNA	Forward	*AAATTATATTGGAGAATAGATTAGATGAGT		58°C
	Reverse	TCCTTATTACTTTCCTCATTAATATACACT		
	Sequencing	ACACTATTCTACAAATCCC	2	

Mouse major satellite DNA	Forward	*TGTGTGTGTATTTATTTTGGAAAGAA		58°C
	Reverse	AACATTTCTAAATATTCCACCTTTTTC		
	Sequencing	CAATATACATTTCTCATTTTTCAC	3	
Mouse LINE1 T^g	Forward	*GGTTGGGGAGGYGGTTTAAGTTATA		60°C
	Reverse	CTACCTATTCCAAAAACTATCAAATTCTT		
	Sequencing 1	AATCCCAAACCAAAATA	2	
	Sequencing 2	CCTATTCAAACTAATTTCCTAAA	2	
Bovine alpha-satellite DNA	Forward	TGTTTTGTTTGGAAGGGGTTT		58°C
	Reverse	*AACTATATTTAAAACCAAAAATTTTTCC		
	Sequencing 1	GTTTGGAAGGGGTTTT	3	
	Sequencing 2	GTGGGTGGTTTTATATT	3	
	Sequencing 3	GGTTTTTTTTTGATAAGAATT	3	
	Sequencing 4	GAAGGGGTAGTTTTT	3	
Bovine testis satellite DNA	Forward	TTGGGTTTGGTGTATTGGAAGA		58°C
	Reverse	*ACTCCACCCCTATAAATACAAT		
	Sequencing 1	GTAGGGTATTTTTGATTTTAGA	2	
	Sequencing 2	GGGTTGAGGTATGGAA	5	
	Sequencing 3	GGAATTTGGGGTTTTTT	2	
Marmoset α-satellite DNA	Forward	GGTTTTTTTAGGAGTTGGAT		57°C
	Reverse	*AAAATTTCCAACTATTTCTTAAATACCT		
	Sequencing 1	GGGTGAATTGGAATAA	2	
	Sequencing 2	TGAGAAATATTTGTTTTTAAAATTA	2	
	Sequencing 3	GGTTTTTAATGTGTGTATTTAATTT	1	

[a] Primers indicated by a star are biotinylated at the 5' end.
[b] Outer primers for nested PCR are only necessary, when working with very small amounts of DNA (equivalent to 10 sperm).

Primers adopted from the literature.
[c] Raval, A., Sridhar, K. J., Patel, S., Turnbull, B. B., Greenberg, P. L., & Mitchell, B.S. (2012). Reduced rRNA expression and increased rDNA promoter methylation in CD34+ cells of patients with myelodysplastic syndromes. *Blood, 120*, 4812-4818.
[d] Teschler, S., Gotthardt, J., Dammann, G., & Dammann, R.H. (2016). Aberrant DNA methylation of rDNA and PRIMA1 in borderline personality disorder. *International Journal of Molecular Sciences*, *17*, E67.
[e] Yang, A. S., Estécio, M. R., Doshi, K., Kondo, Y., Tajara, E. H., & Issa, J. P. (2004). A simple method for estimating global DNA methylation using bisulfite PCR of repetitive DNA elements. *Nucleic Acids Research*, *32*, e38.
[f] Shiao, Y. H., Leighty, R. M., Wang, C., Ge, X., Crawford, E. B., Spurrier, J. M., … Anderson, L.M. (2012). Ontogeny-driven rDNA rearrangement, methylation, and transcription, and paternal influence. *PloS One*, *6*, e22266.
[g] Walter, M., Teissandier, A., Pérez-Palacios, R., & Bourc'his, D. (2016). An epigenetic switch ensures transposon repression upon dynamic loss of DNA methylation in embryonic stem cells. *Elife*, *5*, e11418.

TABLE S3 Correlation between single CpG methylation (in human sperm rDNA, α-satellite DNA and LINE1 elements) and donor's age

Sperm cohort 1 (N = 186)

	**rDNA upstream control element**		**rDNA promoter**		**18S rDNA**		**28S rDNA**		**α-satellite DNA**		**LINE1**	
	Pearson's r	*p* value	Pearson's r	*p* value	Pearsons'r	*p* value	Pearson's r	*p* value	Pearson's r	*p* value	Pearson's r	*p* value
CpG1	0.34	<0.0001	0.57	<0.0001	0.31	<0.0001	0.49	<0.0001	0.30	<0.0001	0.22	0.003
CpG2	0.46	<0.0001	0.59	<0.0001	0.24	0.002	0.43	<0.0001	0.26	0.001	0.31	<0.0001
CpG3	0.60	<0.0001	0.51	<0.0001	0.34	< 0.0001	0.38	<0.0001	0.29	<0.0001	0.30	<0.0001
CpG4	0.41	<0.0001	0.54	<0.0001	0.34	< 0.0001	0.45	<0.0001	0.28	<0.0001	0.27	<0.0001
CpG5	0.50	<0.0001	0.60	<0.0001	0.46	< 0.0001	0.49	<0.0001				
CpG6	0.65	<0.0001	0.60	<0.0001	0.34	< 0.0001	0.43	<0.0001				
CpG7	0.63	<0.0001	0.45	<0.0001	0.43	< 0.0001	0.29	<0.0001				
CpG8	0.50	<0.0001	0.60	<0.0001	0.40	<0.0001	0.40	<0.0001				
CpG9	0.54	<0.0001	0.62	<0.0001			0.43	<0.0001				
CpG10	0.60	<0.0001					0.48	<0.0001				
CpG11	0.56	<0.0001										
CpG12	0.59	<0.0001										
CpG13	0.48	<0.0001										
CpG14	0.65	<0.0001										
CpG15	0.58	<0.0001										
CpG16	0.56	<0.0001										
CpG17	0.48	<0.0001										
CpG18	0.43	<0.0001										

CpG19	0.46	<0.0001										
CpG20	0.27	<0.0001										
CpG21	0.30	<0.0001										
CpG22	0.28	<0.0001										
CpG23	0.32	<0.0001										
CpG24	0.45	<0.0001										
CpG25	0.55	<0.0001										
CpG26	0.54	<0.0001										

Sperm cohort 2 (N = 109)

	**rDNA UCE**		**rDNA promoter**		**18S rDNA**		**28S rDNA**		**α-satellite DNA**		**LINE1**	
	Pearson's r	*p* value	Pearson's r	*p* value	Pearsons'r	*p* value	Pearson's r	*p* value	Pearson's r	*p* value	Pearson's r	*p* value
CpG1	0.44	<0.0001	0.58	<0.0001	0.32	0.002	0.46	<0.0001	0.26	0.013	0.27	0.019
CpG2	0.52	<0.0001	0.60	<0.0001	0.30	0.004	0.42	<0.0001	0.29	0.005	0.35	0.002
CpG3	0.55	<0.0001	0.57	<0.0001	0.37	<0.0001	0.48	<0.0001	0.31	0.002	0.24	0.038
CpG4	0.44	<0.0001	0.58	<0.0001	0.32	0.001	0.44	<0.0001	0.26	0.011	0.27	0.029
CpG5	0.43	<0.0001	0.53	<0.0001	0.41	<0.0001	0.43	<0.0001				
CpG6	0.62	<0.0001	0.46	<0.0001	0.32	0.002	0.45	<0.0001				
CpG7	0.59	<0.0001	0.42	<0.0001	0.37	<0.0001	0.31	0.002				
CpG8	0.50	<0.0001	0.64	<0.0001	0.42	<0.0001	0.35	0.001				
CpG9	0.53	<0.0001	0.59	<0.0001			0.39	<0.0001				
CpG10	0.55	<0.0001					0.45	<0.0001				
CpG11	0.52	<0.0001										
CpG12	0.53	<0.0001										
CpG13	0.50	<0.0001										

CpG14	0.50	<0.0001					
CpG15	0.53	<0.0001					
CpG16	0.50	<0.0001					
CpG17	0.46	<0.0001					
CpG18	0.50	<0.0001					
CpG19	0.39	<0.0001					
CpG20	0.27	0.008					
CpG21	0.23	0.028					
CpG22	0.27	0.008					
CpG23	0.28	0.006					
CpG24	0.49	<0.0001					
CpG25	0.52	<0.0001					
CpG26	0.45	<0.0001					

TABLE S4 Primers for deep bisulfite sequencing of human rDNA

**Assay**	**Primer**	**Sequence (5’-3’)***	**Amplicon length**	**Variant**	**Number of CpGs**	**Annealing temperature**
rDNA region 1 (external transcribed spacer)	Forward	GGAGTTAGYGGGGTGGGGTTGT	272 bp	A/G	38	56°C
	Reverse	ACTAAAAAAATTAAACCTCC				
	Amplified sequence	GGAGCCAGCGGGGTGGGGTTGTCGCGGCCGCCCCGGGCGCCCGCAGCGGAGAGCGCACGGGGGCACGGTGGCCCTCGCCGCCTTCCCCGCCGCCCCCGGGTGGGTCAGAGACCCGGACCCGGGCCGGCACCGGGAGTCGGGACGCTCGGACGCGCGAGAGAA/GCAGCAGGCCCGCGGGCCCCGGCAGGCGGCTCAAGCAGGAGCGCGGCCGGCTAGCCGGGTCACCGGTAGGCCAGAGCCCCGCGCGCATCCGGAGGCCCAACCTCTCCAGCG				
rDNA region 2 (upstream control element and core promoter)	Forward	TATTYGGAGGTTTAATTTTTTTAG	235 bp	A/G	25	56°C
	Reverse	TATATCCTAAAATTAACCAAAAAACCCC				
	Amplified sequence	GGAGGCCCAACCTCTCCAGCGACAGGTCGCCAGAGGACAGCGTGTCAGCAATAACCCGGCGGCCCAAAATGCCGACTCGGAGCGAAAGATATACCTCCCCCGGGGCCGGGAGGTCGCGTCACCGACCACGCCGCCGGCCCAGGCA/GACGCGCGACACGGACACCTGTCCCCAAAAACGCCACCATCGCAGCCACACACGGAGCGCCCGGGGCCCTCTGGTCAACCCCAGGACACAC				

* The A/G variant in the amplified sequence is underlined.

TABLE S5 Correlations between donor's age and mean repeat methylation in different animal models

Region	CpG number	Correlation coefficient	*p* value
Mus musculus (N = 80)			
rDNA spacer promoter	14	Pearson's r = 0.19	0.09
rDNA core promoter	7	Pearson's r = 0.23	0.044
18S rDNA	8	Pearson's r = 0.25	0.024
28S rDNA	10	Pearson's r = 0.36	0.001
Mouse minor satellite	2	Pearson's r = 0.57	< 0.0001
Mouse major satellite	3	Pearson's r = 0.24	0.037
Mouse LINE1-T	4	Pearson's r = 0.27	0.016
Bos taurus (N = 36)*			
18S rDNA	8	Spearman's rho = 0.45	0.006
28S rDNA	10	Spearman's rho = 0.32	0.058
Bovine alpha-satellite	12	Spearman's rho = 0.62	< 0.0001
Bovine testis satellite I	9	Spearman's rho = 0.76	< 0.0001
Callithrix jacchus (N = 16)			
18S rDNA	8	Spearman's rho = 0.02	0.9
28S rDNA	10	Spearman's rho = 0.22	0.4
α-satellite DNA	5	Spearman's rho = 0.63	0.009

* Including samples from 9 bulls at three different ages and 3 bulls at two different ages.

TABLE S6 Sperm methylation of orthologous regions in 18S and 28S rDNA in four analyzed species

	**Methylation (%)** Mean ± SD[a] [range]	**Methylation increase (%) in 10% of lifespan**[b]
**18S rDNA**		
Human (N = 295)	14.2±3.4 [8.1-26.4]	1.83
Marmoset (N = 16)	22.1±6.9 [10.3-36.9]	0.25
Bovine (N = 36)	5.8±2.0 [2.8-12.1]	0.35
Mouse (N = 80)	18.1±3.3 [11.5-27.3]	0.81
**28S rDNA**		
Human (N = 295)	11.5±2.8 [5.7-21.4]	1.73
Marmoset (N = 16)	18.2±4.7 [10.6-27.8]	0.49
Bovine (N = 36)	8.1±2.1 [4.5-12.3]	0.41
Mouse (N = 80)	15.5±3.6 [8.2-25.1]	1.28

[a] SD = standard deviation.
[b] Lifespan is 80 years for humans, 12 years for marmoset, 20 year for bull, and 28 months for mouse.

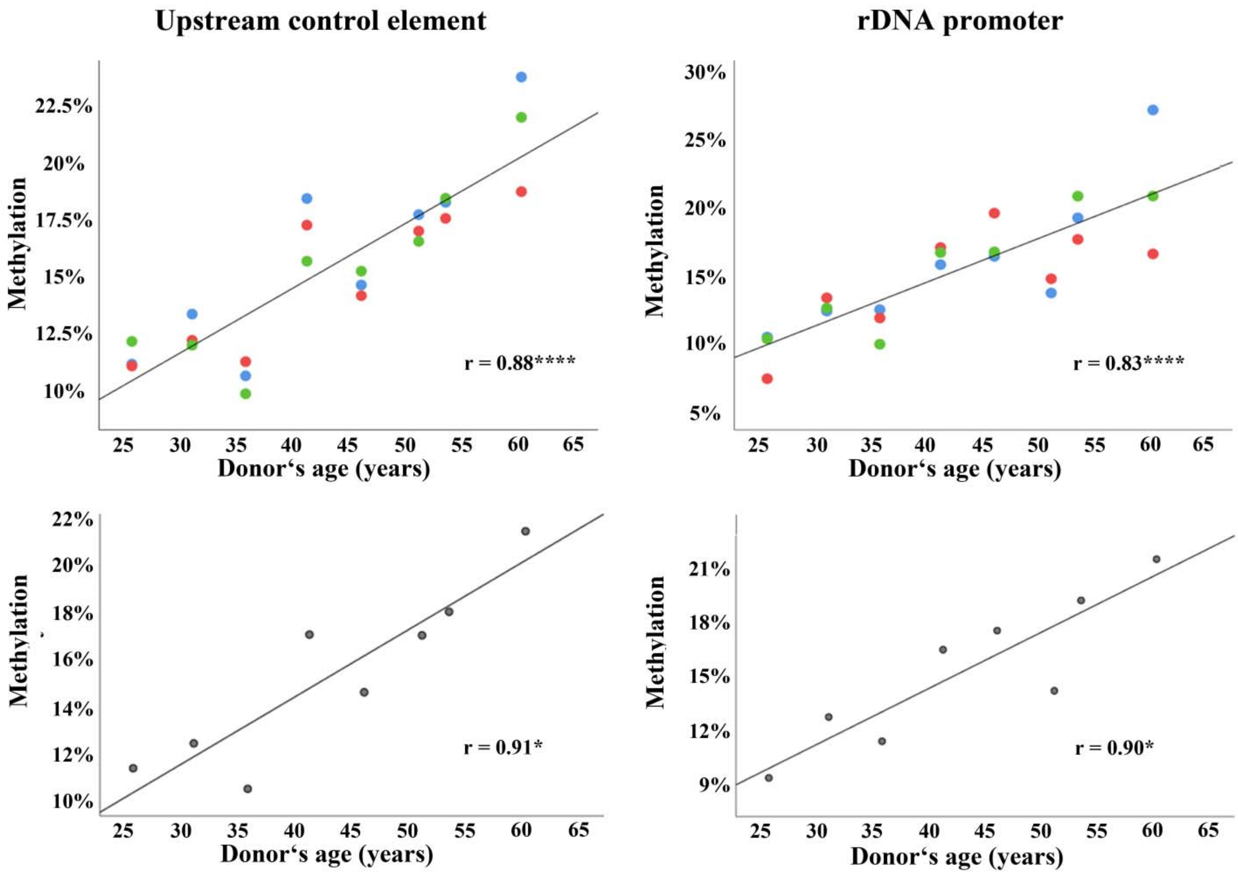

FIGURE S1 rDNA methylation analysis of DNA aliquots equivalent to 10 human sperm. Scatter plots show the correlation between donor's age (x-axis in years) and mean methylation (y-axis in %) of the UCE (26 CpGs) and rDNA promoter (9 CpGs).. DNA aliquots of 8 donors covering an age range from 25 to 60 years with 5-year gaps were analyzed by bisulfite pyrosequencing in triplicates. The blue, green and red dots in the upper diagrams represent the three measurements for each sample; the black dots in the lower diagrams represent the mean of triplicate measurements. Pearson's correlations were used for statistical analysis of the 24 measurements as well as for the means of 8 triplicates. **** indicates $p < 0.0001$ and * $p < 0.02$.

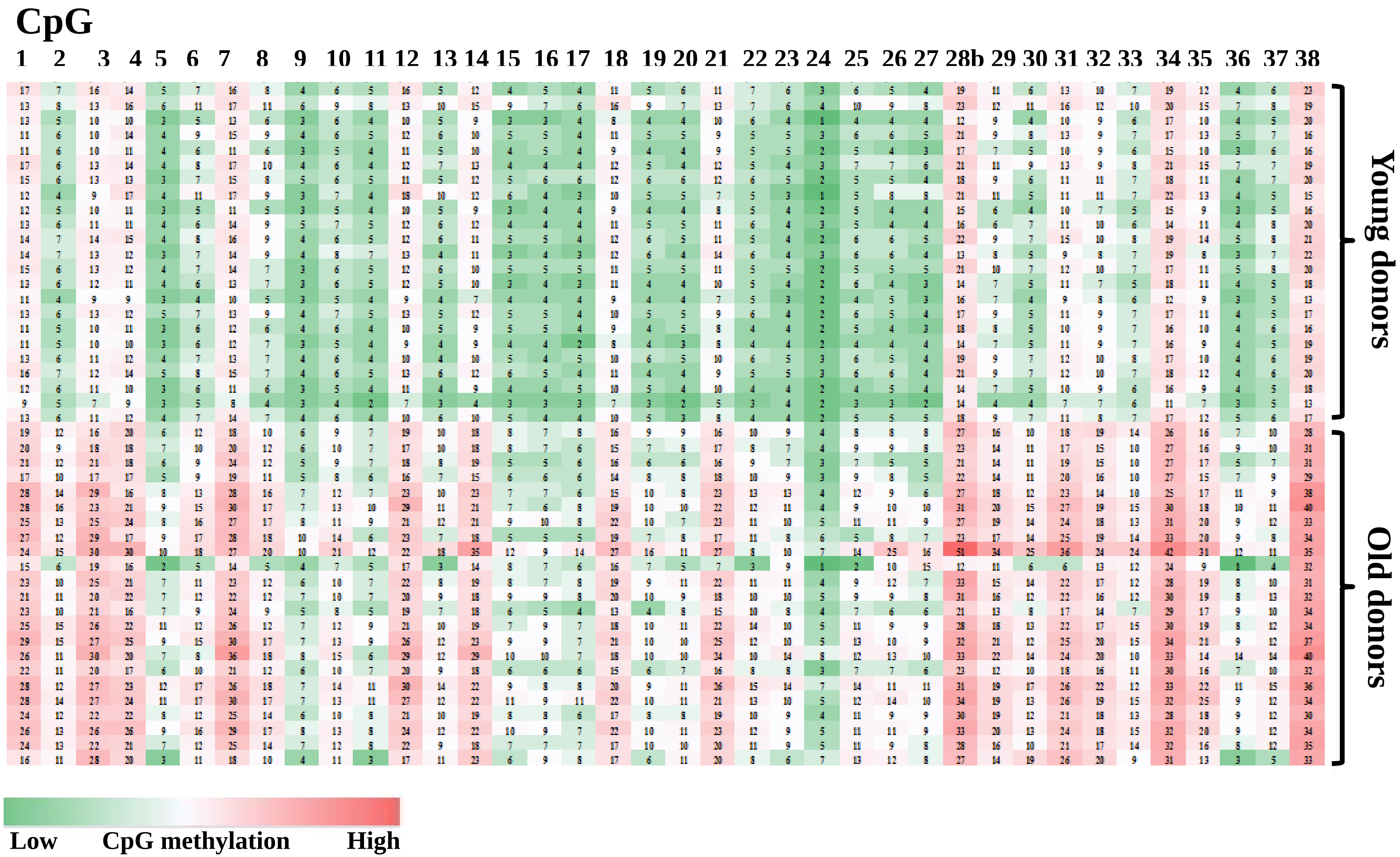

FIGURE S2A Single CpG methylation of the A variant of human rDNA region 1 (in the external transcribed spacer). Each row on the y-axis represents an individual sperm sample, 23 from young donors and 23 from old donors. The x-axis represents CpGs 1-38. The number within each box indicates the methylation percentage of a given CpG in a given sample, determined by deep bisulfite sequencing.

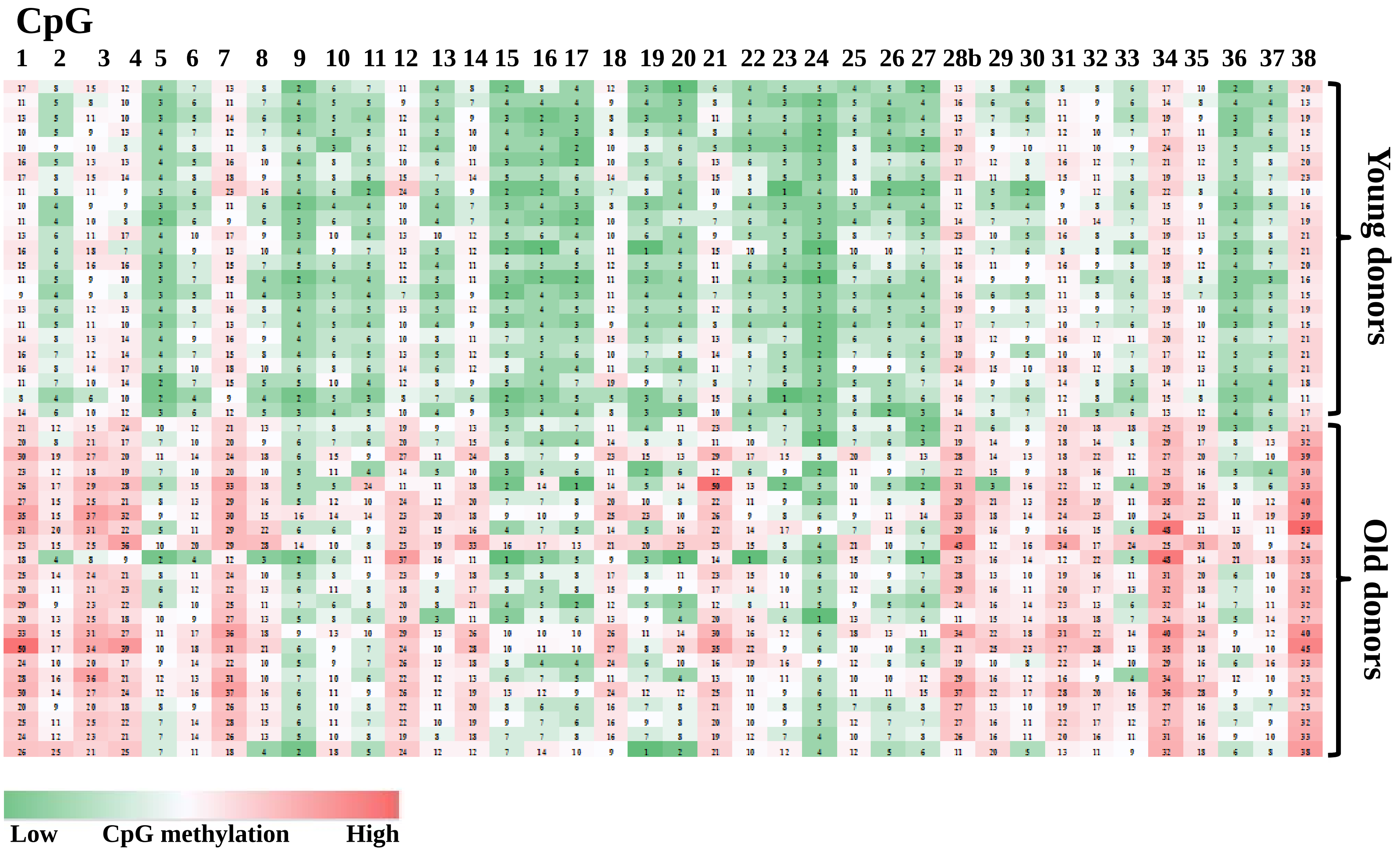

FIGURE S2B Single CpG methylation of the G variant of human rDNA region 1 (in the external transcribed spacer). Each row on the y-axis represents an individual sperm sample, 23 from young donors and 23 from old donors. The x-axis represents CpGs 1-38. The number within each box indicates the methylation percentage of a given CpG in a given sample, determined by deep bisulfite sequencing.

CpG

Donors	1	2	3	4	5	6	7	8	9	10	11	12	13	14	15	16	17	18	19	20	21	22	23	24	25
Young donors	17	5	11	9	4	5	14	7	14	6	10	12	16	13	17	24	31	11	14	14	3	11	7	3	11
	16	7	14	16	11	16	28	9	37	17	13	16	22	18	26	35	40	11	13	14	3	26	10	5	28
	16	5	8	4	2	3	7	4	10	5	6	8	14	9	12	15	26	8	12	14	3	8	6	3	9
	13	5	11	7	6	7	15	4	21	5	8	11	15	14	9	25	28	8	10	11	2	15	6	4	23
	11	4	9	6	3	6	14	5	20	10	6	9	15	12	14	23	27	7	11	10	2	13	7	5	18
	14	5	8	6	4	4	15	6	17	6	6	9	12	10	12	20	27	10	12	12	2	12	6	5	15
	16	6	9	6	4	6	12	5	15	8	8	10	16	14	15	21	29	11	13	14	3	13	7	5	15
	13	5	12	11	7	11	27	6	30	14	13	11	12	14	18	25	39	12	18	15	3	16	10	6	30
	12	4	9	5	2	3	7	3	9	6	6	7	11	9	10	12	20	5	8	10	3	8	3	2	11
	19	8	12	9	6	8	14	7	19	11	11	12	17	13	17	26	31	10	11	15	4	14	7	4	16
	19	6	14	14	11	14	27	9	27	16	12	17	23	21	24	31	40	14	16	17	3	21	6	5	25
	19	7	12	7	4	7	13	6	19	8	6	10	15	8	15	20	31	10	13	14	3	14	10	6	16
	15	5	9	5	3	5	9	5	12	7	8	10	16	15	14	21	28	10	12	14	3	11	6	4	14
	14	4	8	6	3	4	9	4	12	9	9	9	11	11	11	13	20	8	9	13	2	7	5	3	8
	14	5	9	6	3	5	11	5	15	7	8	10	15	12	15	21	29	10	12	14	3	11	6	4	13
	19	4	10	9	8	13	29	9	36	14	12	17	24	20	26	31	38	13	17	12	2	21	7	4	25
	17	7	11	8	3	7	12	5	17	9	8	12	16	12	16	21	27	9	13	15	3	13	8	4	15
	13	5	8	5	2	5	9	4	13	7	6	9	15	11	13	18	26	8	11	12	2	9	6	3	13
	13	4	8	6	4	6	11	5	15	7	7	7	16	13	14	19	28	8	12	13	3	13	7	4	15
	13	5	9	8	5	8	10	4	16	8	7	10	12	11	12	17	21	8	10	10	2	11	5	4	12
	8	4	7	6	4	5	13	3	12	5	3	3	8	6	10	17	19	5	5	7	1	15	6	3	19
	15	6	9	9	7	8	15	5	18	10	10	13	16	14	17	22	30	8	10	12	2	13	7	5	15
Old donors	23	8	12	13	5	9	23	11	28	17	16	15	27	20	26	35	39	18	20	22	5	21	13	8	20
	23	10	15	10	9	15	15	7	21	6	10	14	19	12	25	27	32	12	20	21	5	17	9	7	16
	36	13	18	8	5	8	15	10	16	10	15	17	31	18	26	29	52	27	29	31	6	16	14	8	16
	31	12	18	13	7	14	20	13	24	18	16	22	33	20	35	36	49	24	27	31	8	23	16	9	23
	37	15	22	24	21	22	26	19	35	26	25	32	34	33	41	51	55	34	32	28	11	29	20	11	29
	50	25	40	41	34	36	51	28	54	34	36	38	48	35	56	61	73	50	52	47	13	51	33	17	51
	25	10	14	15	9	14	25	15	35	15	11	15	27	24	25	35	45	17	24	23	5	24	15	8	30
	35	13	18	8	6	8	16	10	19	11	16	20	35	21	29	31	52	27	30	32	6	17	13	8	18
	24	10	12	8	6	7	16	10	18	10	10	13	22	14	24	24	37	15	20	19	3	12	13	7	18
	28	10	13	9	3	7	10	11	21	14	13	18	26	17	23	26	40	18	22	20	4	15	12	8	20
	26	11	15	11	5	11	20	10	25	13	14	19	29	23	27	36	49	22	22	23	5	20	14	9	29
	30	13	21	15	11	15	28	11	26	14	15	18	28	24	30	41	54	22	25	25	5	27	16	10	30
	29	11	16	7	4	7	12	8	18	12	14	17	28	16	24	27	43	19	24	27	5	13	12	6	14
	39	22	30	28	18	27	40	25	51	25	21	24	37	27	31	43	59	31	37	39	10	33	23	17	41
	36	14	23	17	11	14	30	16	33	17	21	24	39	28	37	46	63	31	34	36	8	28	18	10	31
	41	17	23	14	10	15	27	17	33	20	27	31	44	30	41	45	66	37	38	41	10	24	18	11	27
	56	36	51	49	48	45	52	36	71	62	69	68	71	60	74	76	82	53	56	58	11	54	30	20	50
	33	13	20	21	18	19	33	19	39	16	24	23	39	26	36	42	57	24	28	27	9	32	17	13	34
	30	12	22	21	15	20	38	17	43	22	22	26	38	28	41	53	67	31	30	30	7	33	17	11	38
	25	9	13	9	5	8	14	7	20	12	12	15	26	20	22	29	43	18	22	23	4	16	11	8	20
	26	11	15	11	6	11	20	9	23	13	15	18	27	19	24	30	44	17	22	24	5	19	13	7	24
	28	12	15	9	5	8	15	9	23	14	15	18	31	20	26	31	45	19	25	27	6	18	14	8	21

Low CpG methylation High

FIGURE S2C Single CpG methylation of the A variant of human rDNA region 2 (in the upstream control element and core promoter). Each row on the y-axis represents an individual sperm sample, 23 from young donors and 23 from old donors. The x-axis represents CpGs 1-25. The number within each box indicates the methylation percentage of a given CpG in a given sample, determined by deep bisulfite sequencing.

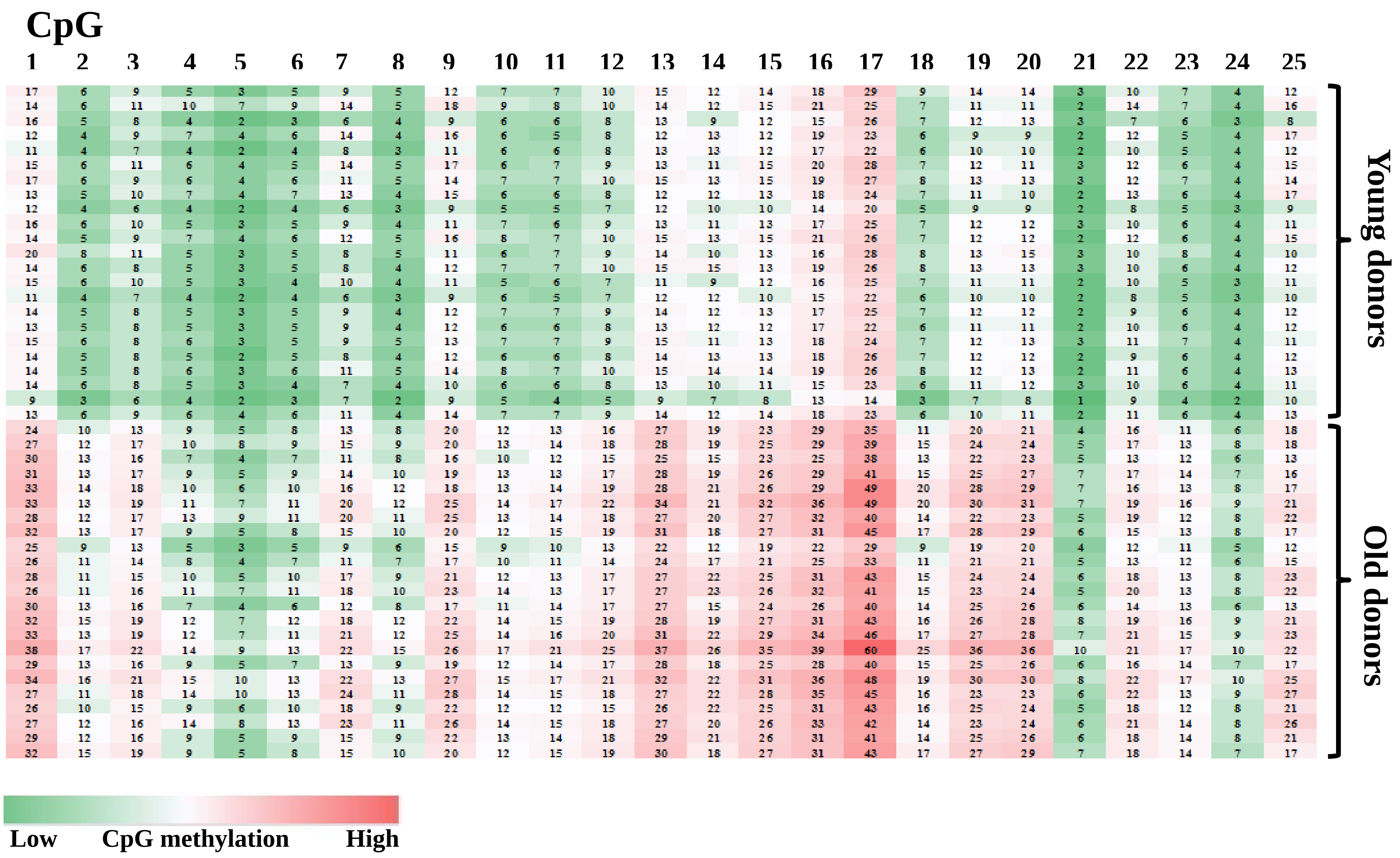

FIGURE S2D Single CpG methylation of the G variant of human rDNA region 2 (in the upstream control element and core promoter). Each row on the y-axis represents an individual sperm sample, 23 from young donors and 23 from old donors. The x-axis represents CpGs 1-25. The number within each box indicates the methylation percentage of a given CpG in a given sample, determined by deep bisulfite sequencing.

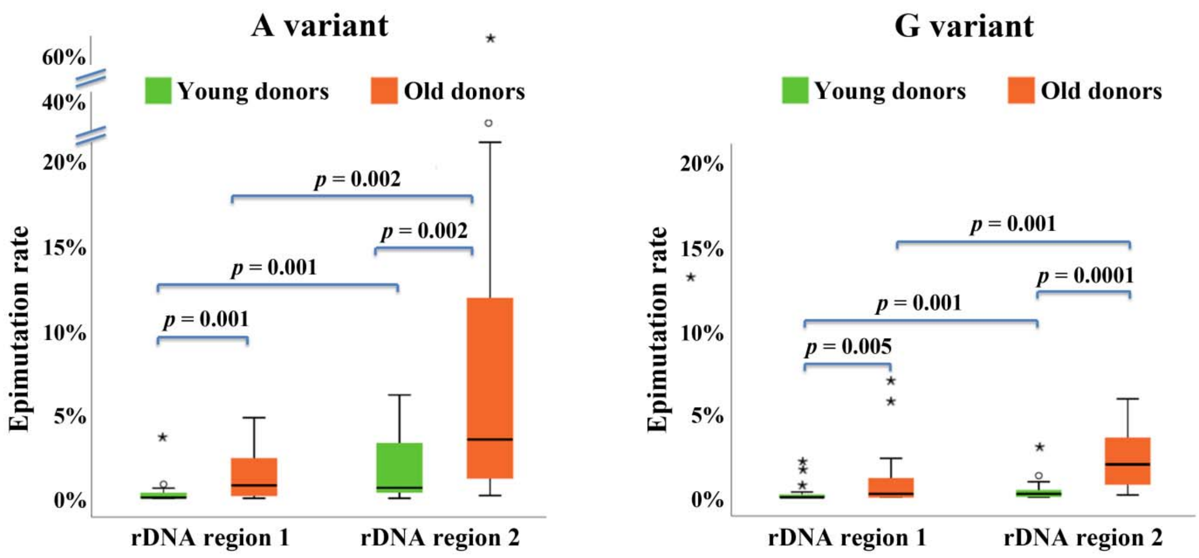

FIGURE S3 Box plots presenting the epimutation rates (ERs) of alleles carrying the A versus the G variant in rDNA regions 1 (representing the external transcribed spacer) and 2 (upstream control element and core promoter). The bottom and the top of the box represent the 25th and 75th percentile, respectively. The median is represented by a horizontal line. Bars extend from the boxes to at most 1.5 times the height of the box. Outliers are indicated by an open circle and extreme outliers by a star symbol. The *p* values indicate significant differences of the ERs between 23 young versus 23 old donors and between rDNA region 1 versus 2, respectively. Mann-Whitney U tests were used for group comparisons.

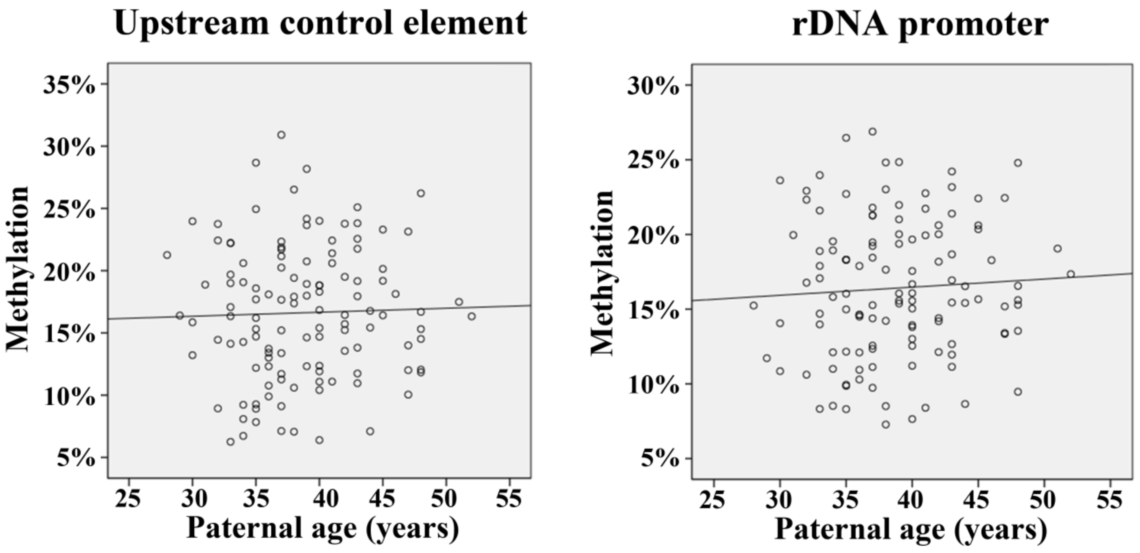

FIGURE S4 Methylation of the rDNA upstream control element (UCE) and promoter in human fetal cord blood. Each dot represents one of 121 cord blood samples. The y-axis indicates mean methylation (in %) of 26 CpG and 9 CpG sites in the UCE and promoter, respectively. The x-axis indicates the age of the father (in years) at conception. There is no significant correlation (Pearson's r = 0.01; p = 0.92 and r = 0.06; p = 0.53, respectively) between cord blood rDNA methylation and paternal age.

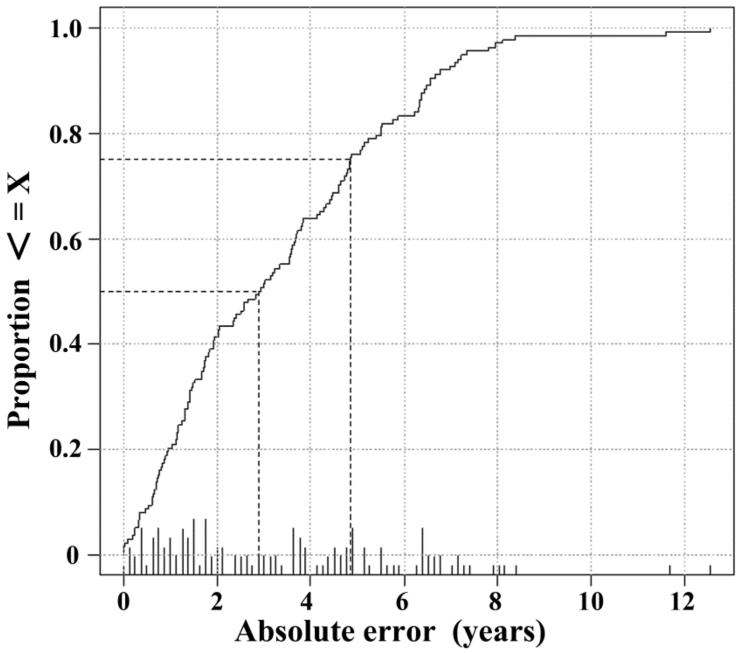

Quantile	Absolute error
0%	0.001
5%	0.310
10%	0.607
15%	0.758
20%	0.981
25%	1.252
30%	1.430
35%	1.726
40%	1.923
45%	2.401
50%	2.911
55%	3.413
60%	3.693
65%	4.202
70%	4.610
75%	4.852
80%	5.469
85%	6.308
90%	6.570
95%	7.242
100%	12.546

FIGURE S5 Distribution of absolute error in the test data set. In total 154 test samples were analyzed. The cumulative distribution (i.e. the fraction of values < = x) is plotted on the y-axis versus the absolute error in years on the x-axis. Reference lines (dotted) are given for the 50% and 75% quantiles.

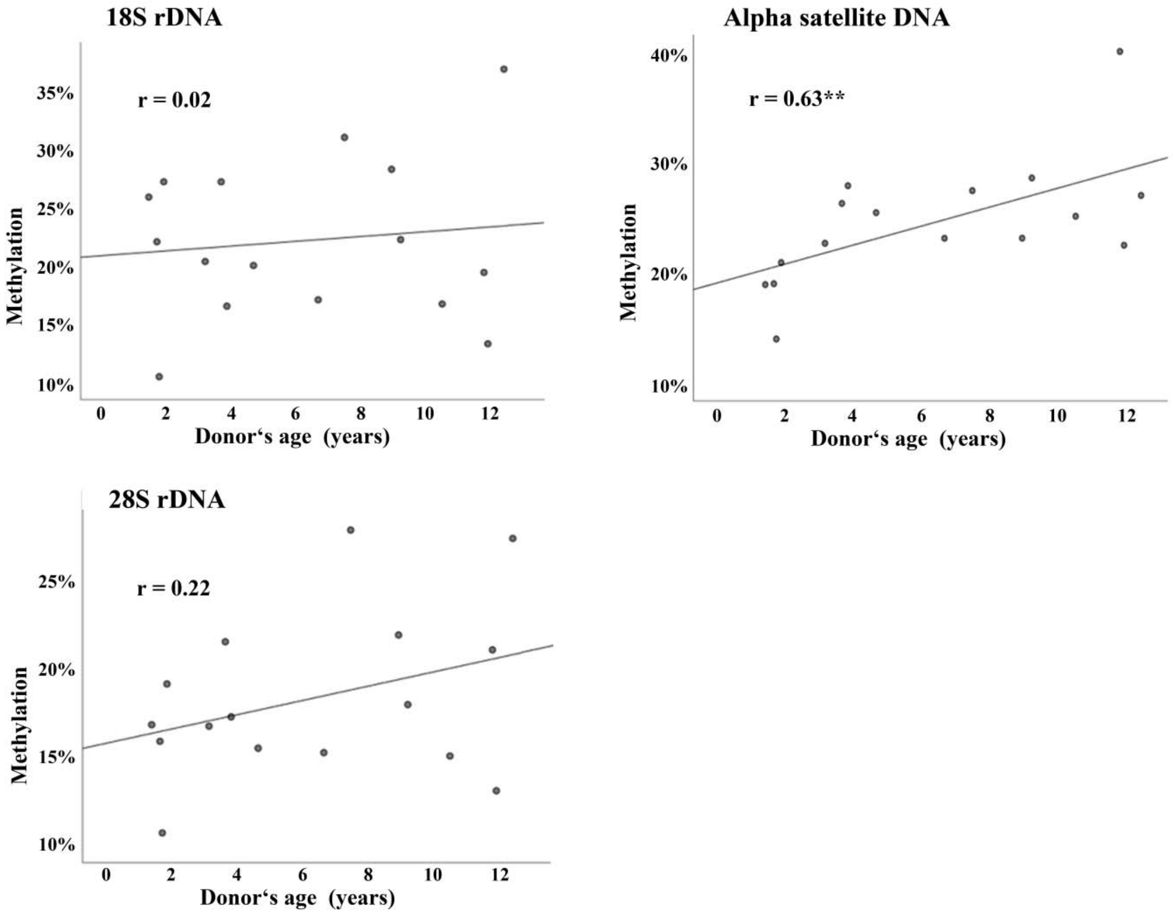

FIGURE S6 Repeat methylation in marmoset sperm increases with donor's age. Scatter plots show positive correlations between donor's age (x-axis in years) and mean methylation (y-axis in %) of 18S rDNA (8 CpGs), 28S rDNA (10 CpGs), and α-satellite DNA (5 CpGs) in marmoset sperm (N = 16). Spearman's correlations were used for statistical analysis of bisulfite pyrosequencing data. ** indicates $p < 0.01$.

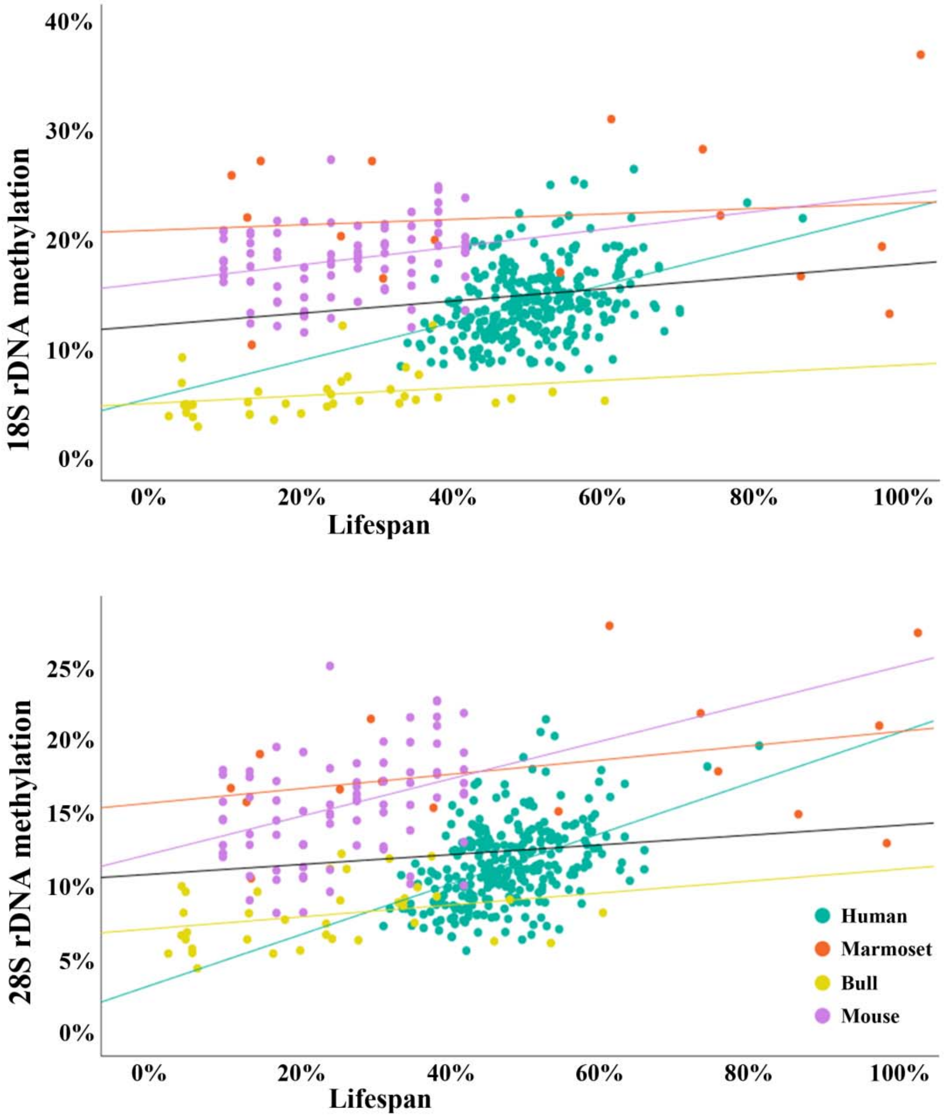

FIGURE S7 Evolutionary conservation of the paternal age effect on sperm methylation of orthologous rDNA regions. Scatter plots show positive correlations between donor's age (x-axis in percentage of lifespan) and mean methylation (y-axis in %) of 18S rDNA (8 CpGs) and 28S rDNA (10 CpGs), respectively, in 80 mouse (purple dots), 36 bull (yellow), 16 marmoset (red), and 295 human (green) sperm samples. Mean methylation of all 427 analyzed samples (indicated by the black regression lines) significantly increased with donor’s age (Pearson's r = 0.20; $p < 0.0001$ for 18S and r = 0.13; $p < 0.01$ for 28S rDNA).
